# CD147 receptor is essential for TFF3-mediated signaling regulating colorectal cancer progression

**DOI:** 10.1038/s41392-021-00677-2

**Published:** 2021-07-14

**Authors:** Hong-Yong Cui, Shi-Jie Wang, Fei Song, Xu Cheng, Gang Nan, Yu Zhao, Mei-Rui Qian, Xi Chen, Jia-Yue Li, Fen-Ling Liu, Yu-Meng Zhu, Ruo-Fei Tian, Bin Wang, Bin Wu, Yang Zhang, Xiu-Xuan Sun, Ting Guo, Xiang-Min Yang, Hai Zhang, Ling Li, Jing Xu, Hui-Jie Bian, Jian-Li Jiang, Zhi-Nan Chen

**Affiliations:** 1 National Translational Science Center for Molecular Medicine and Department of Cell Biology, Fourth Military Medical University, Xi’an, China; 2 Department of Urology, Shenzhen Institute of Translational Medicine, The First Affiliated Hospital of Shenzhen University, Shenzhen Second People’s Hospital, Shenzhen, China; 3 Medical Research Institute, School of Medicine, Wuhan University, Wuhan, China; 4 College of Chemistry and Materials Science, Northwest University, Xi’an, China; 5 National Facility for Protein Science Shanghai, Zhangjiang Lab, Shanghai Advanced Research Institute, Chinese Academy of Sciences, Shanghai, China; 6 Medical Research Center, Southern University of Science and Technology Hospital, Shenzhen, China

**Keywords:** Gastrointestinal cancer, Cell biology

## Abstract

Major gaps in understanding the molecular mechanisms of colorectal cancer (CRC) progression and intestinal mucosal repair have hampered therapeutic development for gastrointestinal disorders. Trefoil factor 3 (TFF3) has been reported to be involved in CRC progression and intestinal mucosal repair; however, how TFF3 drives tumors to become more aggressive or metastatic and how TFF3 promotes intestinal mucosal repair are still poorly understood. Here, we found that the upregulated TFF3 in CRC predicted a worse overall survival rate. TFF3 deficiency impaired mucosal restitution and adenocarcinogenesis. CD147, a membrane protein, was identified as a binding partner for TFF3. Via binding to CD147, TFF3 enhanced CD147-CD44s interaction, resulting in signal transducer and activator of transcription 3 (STAT3) activation and prostaglandin G/H synthase 2 (PTGS2) expression, which were indispensable for TFF3-induced migration, proliferation, and invasion. PTGS2-derived PGE2 bound to prostaglandin E2 receptor EP4 subtype (PTGER4) and contributed to TFF3-stimulated CRC progression. Solution NMR studies of the TFF3-CD147 interaction revealed the key residues critical for TFF3 binding and the induction of PTGS2 expression. The ability of TFF3 to enhance mucosal restitution was weakened by a PTGS2 inhibitor. Blockade of TFF3-CD147 signaling using competitive inhibitory antibodies or a PTGS2 inhibitor reduced CRC lung metastasis in mice. Our findings bring strong evidence that CD147 is a novel receptor for TFF3 and PTGS2 signaling is critical for TFF3-induced mucosal restitution and CRC progression, which widens and deepens the understanding of the molecular function of trefoil factors.

## Introduction

While survival rates in CRC have improved, CRC is still one of the leading causes of cancer-related deaths worldwide. Although evidence suggests that chronic inflammation predisposes colorectal tissue to cancer development and some key factors such as trefoil factors, prostaglandin G/H synthase 2 (PTGS2), and signal transducer and activator of transcription 3 (STAT3) involved in the maintenance of mucosal integrity are implicated in CRC, the underlying molecular mechanisms are only beginning to be elucidated.

Increased expression of TFF peptides is observed after injury in the gastrointestinal tract.^1,2^ Research suggests that TFF3, which is expressed mainly by intestinal goblet cells, plays an important role in protecting the intestinal mucosa from a variety of injuries and is essential for effective mucosal restitution by facilitating cell migration and inhibiting apoptosis and anoikis.^3^ Studies have reported that TFF3 expression is increased during the development and progression of human cancer, including CRC.^4,5^ In addition, in vitro studies, have shown that TFF3 stimulates survival, proliferation, invasion, and metastasis, and inhibits apoptosis of CRC cells,^6–9^ indicating that TFF3 regulates key functional characteristics of oncogenesis. However, the precise molecular mechanism by which TFF3 regulates CRC progression remains unclear.

As a constituent of intestinal mucus, colonic TFF3 was shown to exist mainly as a high molecular weight heteromer and TFF3 can be released from the TFF3-FCGBP heteromer complex.^10^ There is accumulating evidence that TFF3 function is mediated by multiple signaling pathways, including the mitogen-activated protein kinase (MAPK) in gastric cell lines,^11^ phosphatidylinositol-3-kinase-AKT (PI3K-AKT)^7^ and STAT3^8^ in colonic cancer cells, and nuclear factor kappa B (NF-κB) pathways in a rat intestinal epithelial cell line,^12^ however, the mechanisms by which TFF3 activates these signaling pathways are still elusive. As a secreted protein, the hypothesis that TFF3 binds to specific receptors is plausible and corresponds with the finding that TFF3 rapidly activates intracellular signal transduction pathways.

Recently, chemokine receptor types 4 and 7 (CXCR4 and CXCR7) have been reported to mediate TFF3-induced cell migration, but not cell proliferation and MAPK activation in human conjunctiva epithelial cells.^13^ Leucine-rich repeat and immunoglobulin-like domain-containing nogo receptor-interacting protein 2 (LINGO2) was characterized as a binding partner for TFF3, and anti-LINGO2 antibodies blocked TFF3-induced IL-10 production in the human macrophage/monocyte cell line U937. TFF3 binding to LINGO2 reverses tonic inhibition of epidermal growth factor receptor (EGFR) by LINGO2, resulting in heightened EGFR activation.^14^ Other signal transduction data concerning TFF3 remain elusive and mainly result from migration and apoptotic assays, without providing target receptors or mechanistic details.^3^ This lack of knowledge hampers attempts to elucidate the molecular mechanisms underlying the functional role of TFF3 in mucosal restitution and CRC development and indicates that TFF3 may work differently from the canonical receptor-mediated activation of signaling.

This study demonstrates that CD147, a transmembrane protein, is a novel receptor for TFF3. When TFF3 binds to CD147, it promotes interaction between CD147 and CD44 (standard isoform CD44s), resulting in activation of proto-oncogene tyrosine-protein kinase SRC and STAT3 signaling and elevated expression of PTGS2. These events are critical for TFF3-stimulated cell migration, invasion, and proliferation. Blocking the TFF3-CD147 interaction using competitive antibodies or a PTGS2 inhibitor reduces CRC lung metastasis. These findings pave the way for the therapeutic development of gastrointestinal disorders.

## Results

### Augmented TFF3 expression in CRC promotes cancer progression and correlates with poor survival

To identify the potential key players in mucosal restitution and CRC progression, we assessed the gene expression pattern in 163 tissue samples from patients with CRC from TCGA and 517 normal colorectal tissue samples derived from TCGA and the GTEx data portal (Supplementary Fig. 1a) and the differentially expressed genes (DEGs) [adjusted *p*-value < 1e − 20 (*n* = 5820)] were selected for further investigation of their effects on overall survival (Supplementary Fig. 1b, c). Further analysis of the 50 genes with *p* < 0.01 was conducted to identify genes associated with colorectal diseases using the DisGeNET database; nine genes were selected, including TFF3 (Supplementary Fig. 1d). In addition, we compared the mRNA expression of these 9 genes in 26 CRC tissues and paired paracancerous tissues. IL17D was under the detection limit and TFF3 showed the most significant change (Supplementary Fig. 1e). We performed immunohistochemical staining of TFF3 in 75 paired colon cancer and adjacent mucosa tissues. TFF3 expression in the epithelial cells of the cancer tissues was significantly increased compared to that in the adjacent normal mucosa (Supplementary Fig. 1f). We also determined serum TFF3 levels in 188 colon cancer patients and 97 healthy controls (Supplementary Table 1). Serum TFF3 levels were significantly higher in patients with CRC than in healthy controls (Supplementary Fig. 1g) and serum TFF3 level was positively correlated with TFF3 expression in CRC tissues (Supplementary Table 2).

We then determined TFF3 expression and secretion in multiple CRC cell lines (Supplementary Fig. 2). Using in vitro assays, we showed that overexpression of TFF3 in HCT-8 cells with low endogenous TFF3 expression facilitated cell migration (Supplementary Fig. 3a), invasion (Supplementary Fig. 3b), and proliferation (Supplementary Fig. 3c). In addition, the knockout of TFF3 in SW620 cells with high endogenous TFF3 expression showed the opposite effect (Supplementary Fig. 3d–g). Further analysis showed that TFF3 regulated cell cycle progression (Supplementary Fig. 3h) and cyclin A expression (Supplementary Fig. 3i) and had little influence on cell death (Supplementary Fig. 3j).

To test the tumorigenic potential of TFF3 in vivo, we performed a xenograft assay in immunodeficient mice. Tumor progression was increased significantly in mice injected with HCT-8 overexpressing TFF3 (Supplementary Fig. 3k). Furthermore, we used a tail vein injection assay to investigate whether the metastatic ability of tumor cells is influenced by TFF3 in vivo. Sixty days after injection, HCT-8 overexpressing TFF3 formed significantly more metastatic nodules in the lungs than did vector control cells (Supplementary Fig. 3l). Kaplan-Meier analysis showed that high TFF3 expression correlated with poor overall survival (*p* = 0.0289) (Supplementary Fig. 3m). These results suggest that colonic TFF3 may function in tumor initiation or as a neoplastic factor that contributes to CRC progression.

### Loss of TFF3 protects against tumor development in an experimental model of colitis-associated cancer

To further investigate the functional role of TFF3 in colitis-associated cancer (CAC), we generated *Tff3*^−/−^ mice, which appeared to develop normally and were grossly indistinguishable from their heterozygous and wild-type littermates (Supplementary Fig. 4a). *Tff3*^−/−^ and matched wild-type (Wt) mice were exposed to azoxymethane (AOM) plus dextran sodium sulfate (DSS) (Supplementary Fig. 4b). *Tff3*^−/−^ mice showed significantly fewer tumors than Wt mice (Supplementary Fig. 4c). Pathologic analysis showed that nearly 60% of Wt mice developed adenocarcinoma; however, none of the *Tff3*^−/−^ mice developed adenocarcinoma (Supplementary Fig. 4d, e). These findings demonstrated that TFF3 promotes CAC initiation and progression.

### CD147 is identified as a TFF3-binding protein and is indispensable for promoting cancer progression and activating downstream signaling by TFF3

**Fig. 1:**
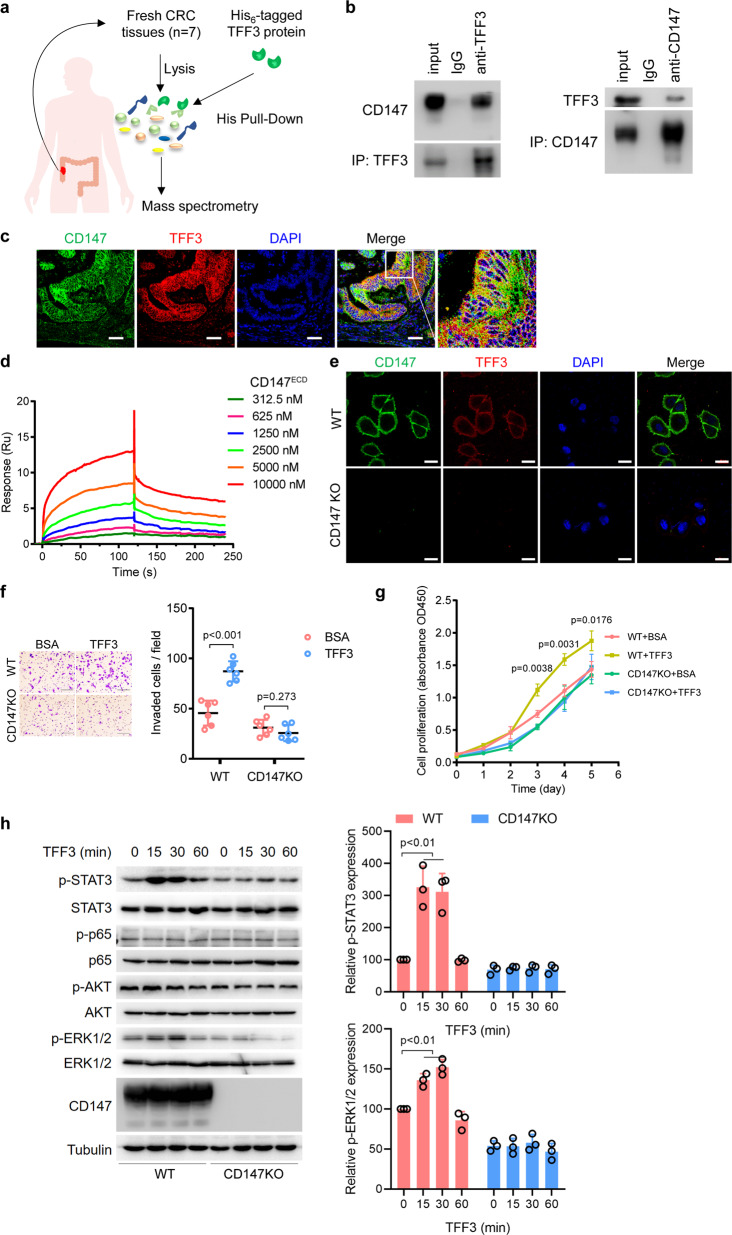
CD147 is identified as a TFF3-binding protein and is indispensable for promoting cancer progression and activating downstream signaling by TFF3. **a** Schematic representation illustrating the strategy of using His_6_-tagged TFF3 to capture interacting proteins from CRC tissues and of characterizing the interacting partner via LC-MS/MS. **b** Western blotting analyses of endogenous TFF3 co-IP with CD147 from CRC tissues. IgG was used as a control antibody. **c** Representative images of immunofluorescence staining of endogenous TFF3 and CD147 in CRC tissues. Scale bar, 50 μm. **d** Biacore diagram of human TFF3 protein bound to CD147^ECD^. The K_D_ values were calculated by the Biacore T200 evaluation software. **e** Representative confocal images of HCT-8 and HCT-8 CD147KO cells incubated with Alexa Fluor™ 555-labeled TFF3. Scale bar, 10 μm. **f** Representative images of HCT-8 and HCT-8 CD147KO cells invading through matrigel-coated transwell inserts toward serum for 24 h. Cells were treated with 0.152 μM of TFF3 or BSA. Scale bar, 50 μm. The graph shows the average number of invaded cells per field. **g** Proliferation curve of HCT-8 and HCT-8 CD147KO cells determined by CCK-8 assays. Cells were treated with 0.152 μM of TFF3 or BSA. Significance relative to the WT+BSA group was determined by a two-tailed Student’s *t*-test. **h** Western blotting analyses of the indicated proteins in HCT-8 or HCT-8 CD147KO cells treated with 0.152 μM of TFF3. Graphs show semi-quantitative analysis of relative p-STAT3 and p-ERK1/2 expression. The *p*-values in **f**–**h** were determined by using two-tailed Student’s *t*-test

To understand the molecular mechanisms by which TFF3 regulates tumor initiation and progression, a His pull-down was employed to capture the proteins interacting with TFF3 (Fig. 1a), where purified His_6_-tagged TFF3 (Supplementary Fig. 5a and Supplementary Table 3) was the bait. Through LC-MS/MS analysis, CD147 emerged as having the highest probability of being a candidate binding partner for TFF3 (Supplementary Table 4). We showed that endogenous TFF3 could form a complex with CD147 in CRC tissues (Fig. 1b) and SW620 cells (Supplementary Fig. 5b). The co-localization of TFF3 and CD147 in CRC tissues was confirmed via immunofluorescence staining (Fig. 1c). The binding of CD147 with TFF3 was further confirmed with CD147-immobilized ELISAs (Supplementary Fig. 5c). In addition, we showed that TFF3 directly interacted with the extracellular domain of CD147 (CD147^ECD^) by using SPR assay with purified proteins (K_D_ = 2.03 ± 0.15 μM, Fig. 1d). TFF1 and TFF2, the other two members of the trefoil factor family that were used as controls in the SPR assay, did not bind to CD147^ECD^ (Supplementary Fig. 5d), indicating that CD147 specifically interacts with TFF3.

To investigate the existence of other TFF3 interactors on the cell surface, HCT-8 or CD147-knockout HCT-8 cells were incubated with fluorescein-labeled TFF3, as shown in Fig. 1e. TFF3 only bound to CD147-expressing cells, indicating that CD147 is the major interactor for TFF3. Mucin has been reported to upregulate TFF3-binding molecules on the cell surface.^15^ Consistent with this report, we confirmed that mucin could promote CD147 cell membrane localization (Supplementary Fig. 5e). These results suggest that CD147 is a specific and major receptor for TFF3 on the surface of CRC cells.

Since TFF3 has been demonstrated to promote cell motility, invasion, and proliferation in cancer cells (Supplementary Fig. 3) and stimulate some signaling pathways, we wondered whether these functions are dependent on the interaction of TFF3 with CD147. A panel of cell lines was treated with TFF3 (Supplementary Fig. 5f). TFF3 promoted migration in the HEK293, SW480, and HCT-8 cell lines, which expressed CD147 (Supplementary Fig. 5g–i). In sharp contrast, TFF3 was unable to increase migration regardless of dose in SW480 CD147KD, HCT-8 CD147KO, and SW1116 cells, which did not express CD147 or in which CD147 was silenced (Supplementary Fig. 5h–j). Moreover, we found that TFF3 failed to promote invasion and proliferation in HCT-8 CD147KO cells (Fig. 1f–g). We then examined downstream signaling and found that TFF3 could stimulate STAT3 and ERK1/2 in a time-dependent manner in CD147-expressing HCT-8 cells, and this stimulatory effect was abolished when CD147 was knocked out (Fig. 1h). Furthermore, we found that TFF3 secretion into the culture supernatant was largely independent of CD147 expression status (Supplementary Fig. 5k). In addition, TFF3-overexpressing HCT-8 cells showed increased STAT3 activity, as well as cell movement, which was abolished when CD147 was knocked out (Supplementary Fig. 5l–m).

We also determined the role of CD147 in migration and proliferation using CD147 knockdown HCT116 cells (Supplementary Fig. 6a). Silence of CD147 led to reduced cell migration and proliferation (Supplementary Fig. 6b, c). Furthermore, we evaluated the role of CD147 in CAC using *Cd147*^f/f^ *Villin*^Cre/+^ mice, we found that intestinal epithelial-specific knockout of *Cd147* reduced tumor development in the AOM/DSS model (Supplementary Fig. 6d–f). These results suggest that CD147 is a functional cell surface receptor for TFF3.

### Defining the TFF3-binding site on CD147 and the key residues

**Fig. 2:**
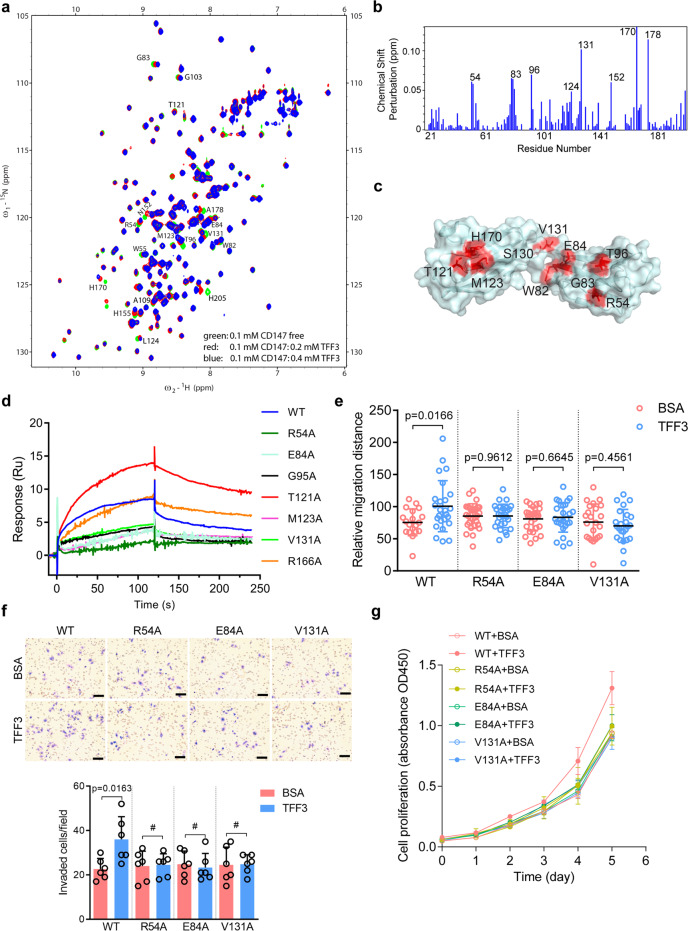
Defining the binding site of TFF3 on CD147 and the key residues. **a** ^15^N-^1^H-HSQC spectra for 0.1 mM CD147^ECD^ were generated in the presence of 0.2 mM (red) or 0.4 mM (blue) TFF3, or the absence of TFF3 (green); the spectra were then superimposed. Residues that show CSPs due to TFF3 binding are labeled. **b** Plot of chemical shift perturbations between CD147^ECD^/TFF3 (ratio 1:4) and free CD147^ECD^. Residues with significant CSPs [Δδ(N–H) > 0.048 ppm] were labeled. **c** TFF3-induced CSPs mapped onto the 3D structure of human CD147^ECD^ (PDB ID: 3B5H, residues 22–203). The colors in the space-filling model correspond to the amplitude of the observed CSPs [red: Δ*δ*(N–H) > 0.035 ppm]. **d** Biacore diagram of human TFF3 protein bound to CD147^ECD^ mutants. **e** Quantification of the migration ability of HCT-8 CD147KO cells transfected with wild-type (WT), R54A, E84A, or V131A CD147 in the presence of 0.152 μM of TFF3 or BSA treatment. **f** Representative images of HCT-8 CD147KO cells invading through matrigel-coated transwell inserts towards serum for 24 h. Cells transfected with WT, R54A, E84A, or V131A CD147 were treated with 0.152 μM of TFF3 or BSA. Scale bar, 50 μm. The graph shows the average number of invaded cells per field. The *p*-values in **e**–**f** were determined by using a two-tailed Student’s *t*-test (^#^*p* > 0.05). **g** Proliferation curve of HCT-8 CD147KO cells determined by CCK-8 assays. Cells transfected with WT, R54A, E84A, or V131A CD147 were treated with 0.152 μM of TFF3 or BSA

We used NMR spectroscopy to elucidate the interaction between CD147 and TFF3. The TFF3-binding site on CD147^ECD^ can be identified by comparing the two-dimensional ^1^H–^15^N heteronuclear single quantum coherence (HSQC) spectra of CD147^ECD^ with or without TFF3 (Fig. 2a). Analysis of the ^1^H–^15^N HSQC spectra from CD147^ECD^ showed that some backbone amide N^1^H and/or N resonances exhibited significant chemical shift perturbations (CSPs) upon TFF3 binding (Fig. 2b, c). By mapping these residues onto the crystal structure of CD147^ECD^, we observed that these residues were spatially clustered and indicated the TFF3-binding site on CD147^ECD^.

To confirm the identity of the TFF3-binding site, we mutated potential key residues in CD147^ECD^ to alanine. As shown in Fig. 2d and Supplementary Fig. 7a, the R54A, E84A, and V131A mutants showed a significantly impaired ability to bind to TFF3; however, these mutations did not have a significant effect on the overall structure of CD147^ECD^ (Supplementary Fig. 7b–d). Sequence analysis revealed that R54 and E84 are evolutionarily conserved in CD147 proteins (Supplementary Fig. 7e).

To verify the structural results and the functional relevance of the identified residues in cells, we treated HCT-8 CD147KO cells expressing wild-type (WT), R54A, E84A, or V131A CD147 with or without TFF3 and found that cells expressing these mutants displayed impaired cell motility (Fig. 2e), invasion (Fig. 2f) and proliferation (Fig. 2g) upon TFF3 treatment compared to cells expressing WT CD147; CD147 membrane localization was not affected by these mutations (Supplementary Fig. 7f). These results demonstrate that the direct interaction between TFF3 and CD147 is responsible for the TFF3-mediated increase in cell motility, invasion, and proliferation.

**Fig. 3:**
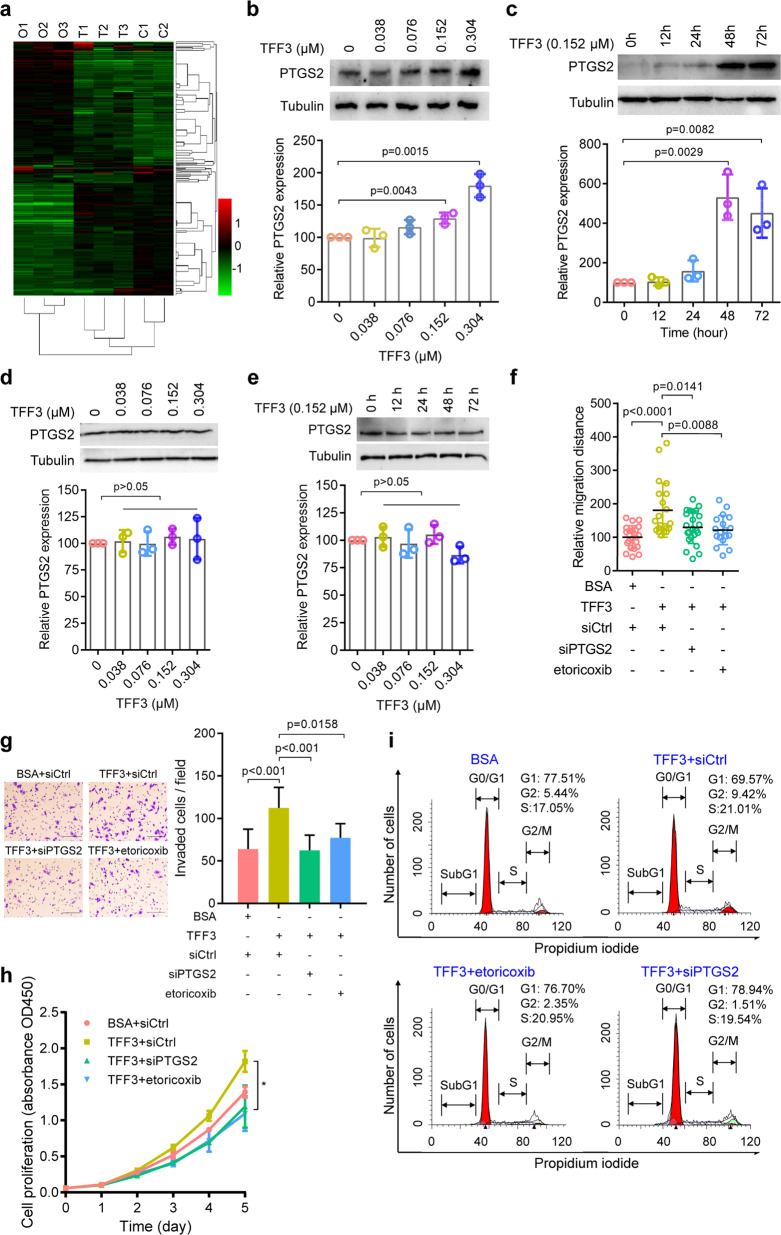
TFF3 promotes migration, invasion, and proliferation via PTGS2. **a** Heatmap of differentially expressed proteins in control (C1–C2), TFF3-treated (T1–T3), and TFF3-overexpressing (O1–O3) HCT-8 cells. **b**–**c** Western blotting analyses of PTGS2 expression in SW480 cells treated with increasing amounts of TFF3 for 48 h (**b**) or following increasing periods of TFF3 treatment (**c**). **d**–**e** Western blotting analyses of PTGS2 expression in HCT-8 CD147KO cells treated with increasing amounts of TFF3 for 48 h (**d**) or following increasing periods of TFF3 treatment (**e**). Graphs in **b**–**e** show semi-quantitative analyses of relative PTGS2 expression. **f** Quantification of cell migration ability of HCT-8 cells treated with 0.152 μM of TFF3 alone or in combination with either siPTGS2 transfection or the PTGS2 inhibitor etoricoxib. **g** Representative images of HCT-8 cells invading through matrigel-coated transwell inserts toward serum for 24 h. Cells were treated with 0.152 μM of TFF3 alone or in combination with either siPTGS2 transfection or the PTGS2 inhibitor etoricoxib. Scale bar, 50 μm. The graph shows the average number of invaded cells per field. The *p*-values in (**b**–**g**) were determined by using a two-tailed Student’s *t*-test. **h** Proliferation curve of HCT116 cells determined by CCK-8 assay. Cells were treated with 0.152 μM of TFF3 alone or in combination with either siPTGS2 transfection or the PTGS2 inhibitor etoricoxib. Significance relative to the TFF3 treatment group was determined by using a two-tailed Student’s *t*-test (*p < 0.05). **i** Flow cytometry analysis of cell cycle progression in HCT-8 cells treated with 0.152 μM of TFF3 alone or in combination with either siPTGS2 transfection or the PTGS2 inhibitor etoricoxib

To identify the downstream effectors of TFF3 in CRC, we analyzed the changes in the expression profiles of protein induced by TFF3 treatment or overexpression in HCT-8 cells. After analyzing the overlap among the three groups, we detected 4230 common proteins and identified 45 differentially expressed proteins between the control and TFF3-treated cells and 233 differentially expressed proteins between the control and TFF3-overexpressing cells with a 1.3-fold change (Fig. 3a, Supplementary Table 5). Among these differentially expressed proteins, 15 were upregulated or downregulated in both types of TFF3-manipulated cells compared with the untreated cells (Supplementary Fig. 8a). The upregulation of PTGS2 and the chaperone activity of bc1 complex-like (CABC1) were further confirmed via western blotting (Supplementary Fig. 8b). In addition, we found that TFF3 promoted PTGS2 protein expression in a dose-dependent and time-dependent manner in both SW480 and HCT-8 CRC cell lines (Fig. 3b, c and Supplementary Fig. 8c, d). In contrast, TFF3 did not induce PTGS2 expression in HCT-8 CD147KO cells (Fig. 3d, e), indicating that CD147 is required for TFF3-induced PTGS2 expression.

Gene expression analysis using GEO data showed that TFF3 expression was highly correlated with PTGS2 expression (Supplementary Fig. 8e). Real-time RT-PCR showed that TFF3 induced *PTGS2* mRNA expression in a time-dependent manner in both SW480 (Supplementary Fig. 8f) and HCT-8 cell lines (Supplementary Fig. 8g) but not in HCT-8 CD147KO cells (Supplementary Fig. 8h). We also found that TFF3 treatment enhanced *PTGS2* reporter activity (Supplementary Fig. 8i). Consistent with the above results, immunohistochemical staining of human CRC tissue microarrays showed that TFF3 expression was strongly correlated with PTGS2 expression (Supplementary Fig. 8j, Supplementary Table 6). TFF3 expression was not correlated with CD147 expression (Supplementary Table 7). As CD147 can be proteolytically cleaved resulting in a shed form of CD147, we determined serum CD147 and found that serum CD147 levels correlated positively with serum TFF3 levels in the CRC patients (*r* = 0.228, *p* = 0.042, Supplementary Table 8).

To confirm that PTGS2 is induced by TFF3 via binding to CD147, we examined HCT-8 CD147KO cells expressing R54A, E84A, V131A, or WT CD147 treated with or without TFF3. We found that TFF3 failed to increase PTGS2 expression in cells expressing mutated receptors (Supplementary Fig. 8k), indicating that the induction of PTGS2 by TFF3 is dependent on the TFF3-CD147 interaction. Furthermore, we showed that PTGS2 expression was increased in a dose-dependent manner in a normal fetal human colon (FHC) cell line (Supplementary Fig. 8l). These results suggest that TFF3 induces PTGS2 expression in both normal and malignant colon cells and that this induction is dependent on the TFF3-CD147 interaction.

Because PTGS2 has been recognized to regulate cell migration, invasion, and proliferation,^16,17^ we investigated whether PTGS2 mediates the effects of TFF3 on these processes. We found that silencing PTGS2 expression using siRNA or selectively inhibiting PTGS2 activity with etoricoxib could negate the impact of TFF3 on cell migration (Fig. 3f, Supplementary Fig. 8m), invasion (Fig. 3g), proliferation (Fig. 3h), and cell cycle progression (Fig. 3i).

As we have shown that TFF3 can enhance CABC1 expression (Supplementary Fig. 8a, b), we ruled out the potential role of CABC1 in TFF3-induced migration, invasion, and proliferation. We then silenced CABC1 expression and found that TFF3 enhanced migration (Supplementary Fig. 8n), invasion (Supplementary Fig. 8o), and proliferation (Supplementary Fig. 8p) regardless of CABC1 expression status in HCT-8 cells.

These results suggest that TFF3 facilitates migration, invasion, and proliferation, mainly by stimulating PTGS2 expression.

### TFF3 induces PTGS2 expression by promoting the interaction between CD147 and CD44s

**Fig. 4:**
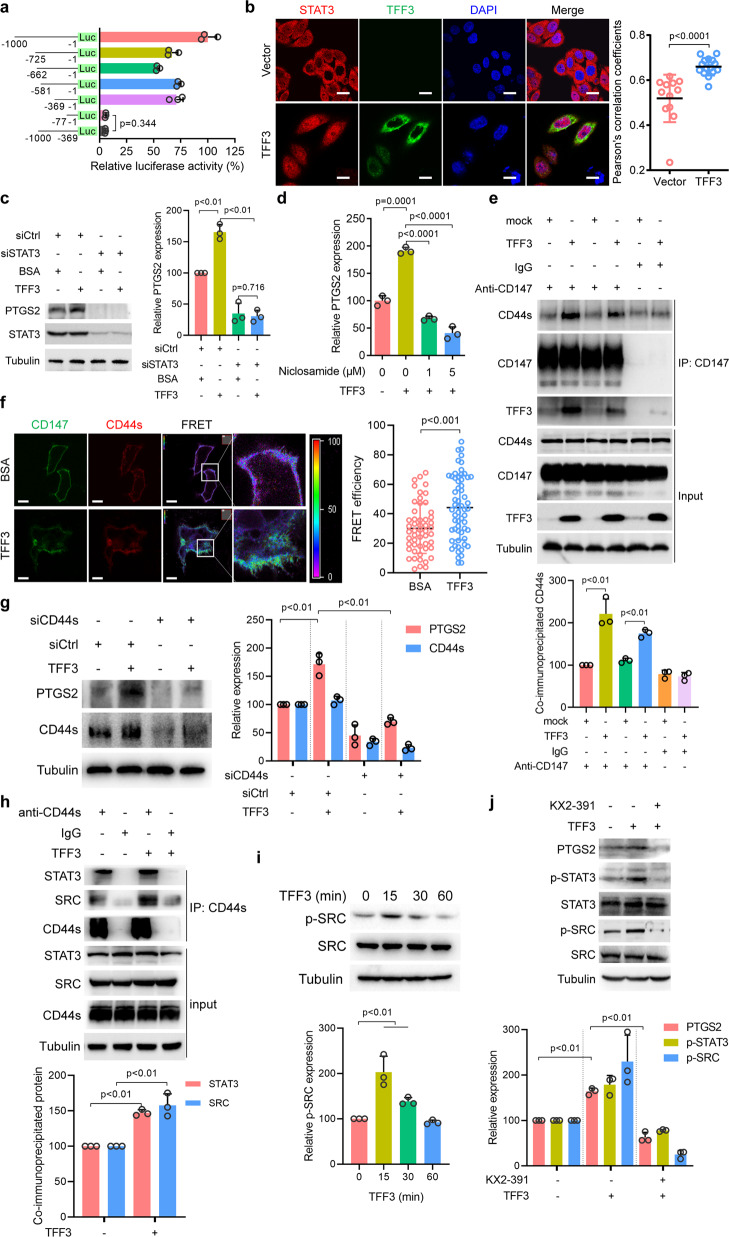
TFF3 induces PTGS2 expression via promoting the interaction between CD147 and CD44s. **a** Schematic representation of the *PTGS2* promoter-reporter constructs. HCT-8 cells were transfected with the indicated constructs. **b** Representative images of immunofluorescence staining of endogenous STAT3 in HCT-8 cells transfected with control plasmid or TFF3. Scale bar, 10 μm. The graph shows Pearson’s correlation coefficients between STAT3 and DAPI. **c** Western blotting analyses of the indicated proteins in HCT-8 cells treated with 0.152 μM of TFF3 alone or in combination with either control siRNA (siCtrl) or siRNA targeting STAT3 (siSTAT3). The graph shows a semi-quantitative analysis of relative PTGS2 expression. **d** qPCR for *PTGS2* normalized to *GAPDH* expression in HCT-8 cells treated with 0.152 μM of TFF3 alone or in combination with niclosamide. **e** Western blotting analyses of endogenous CD147 co-immunoprecipitated with endogenous CD44s in the presence or absence of 0.152 μM of TFF3. IgG was used as a control antibody for immunoprecipitation. The graph shows a semi-quantitative analysis of co-immunoprecipitated CD44s. **f** The interaction between the indicated constructs in the presence or absence of TFF3 was analyzed with fluorescence resonance energy transfer (FRET). The color bar represents the FRET ratio. Scale bar, 10 μm. The graph shows FRET efficiency. **g** Western blotting analyses of the indicated proteins in HCT-8 cells treated with 0.152 μM of TFF3 alone or in combination with either siRNA targeting CD44s (siCD44s) or control siRNA (siCtrl). The graph shows a semi-quantitative analysis of PTGS2 and CD44s expression. **h** Western blotting analyses of endogenous CD44s co-IP with endogenous STAT3 and SRC in the presence or absence of 0.152 μM of TFF3. IgG was used as a control antibody for immunoprecipitation. The graph shows a semi-quantitative analysis of co-immunoprecipitated STAT3 and SRC. **i** Western blotting analyses of SRC activation in HCT116 cells after increasing periods of 0.152 μM of TFF3 treatment. The graph shows a semi-quantitative analysis of p-SRC expression. **j** Western blotting analyses of the indicated proteins in HCT-8 cells treated with 0.152 μM of TFF3 alone or in combination with the SRC inhibitor KX2-391. The graph shows a semi-quantitative analysis of PTGS2, p-STAT3, and p-SRC expression. The *p*-values in **a**–**j** were determined by two-tailed Student’s *t*-test

We showed that TFF3 regulates PTGS2 expression by binding to its receptor CD147. We next tried to identify the intracellular signaling molecules responsible for this regulation. Further analysis using a series of 5’-truncated versions of the *PTGS2* promoter showed that the promoter region between −369 and −77 was indispensable for the basal transcription of *PTGS2* in CRC cells (Fig. 4a). Sequence analysis identified one NF-κB binding site and two STAT3 binding sites in the promoter between −369 and −77 (Supplementary Fig. 9a). Detailed analysis showed that mutation of the NF-κB binding site had a minimal effect on reporter activity, whereas mutation of the STAT3 binding sites, especially the first binding site, decreased reporter activity (Supplementary Fig. 9b), indicating that *PTGS2* transcription was primarily regulated by STAT3, not NF-κB. Electrophoretic mobility shift assay (EMSA) and ChIP assay confirmed that TFF3 increased the binding of STAT3 to the PTGS2 promoter in cells expressing WT CD147 (Supplementary Fig. 9c, d). These results were further confirmed by overexpression or siRNA-mediated knockdown of STAT3 in HCT-8 cells, which resulted in increased or decreased PTGS2 expression, respectively (Supplementary Fig. 9e, f). In addition, inhibiting STAT3 signaling with niclosamide or WP1066 blocked PTGS2 expression in a dose-dependent manner (Supplementary Fig. 9g). Though TFF3 activated ERK1/2 signaling (Fig. 1h), inhibiting ERK1/2 signaling with GDC-0623 had little effect on PTGS2 expression (Supplementary Fig. 9h).

We also found that overexpression of TFF3 promoted STAT3 nuclear localization (Fig. 4b and Supplementary Fig. 9i). Mutation of the STAT3 binding sites impaired the TFF3-induced increase in reporter activity (Supplementary Fig. 9b). Furthermore, knockdown of STAT3 offset TFF3-induced PTGS2 expression (Fig. 4c). The TFF3-induced expression of PTGS2 in CRC cells could be abolished by the STAT3 inhibitor niclosamide (Fig. 4d, Supplementary Fig. 9j). To provide further evidence, we isolated primary carcinoma cells from two human CRC specimens and found that TFF3 induced PTGS2 expression in a dose-dependent manner, which could be blocked by niclosamide (Supplementary Fig. 9k). These results suggest that STAT3 signaling mediates PTGS2 expression stimulated by TFF3.

Since TFF3 binds to the ectodomain of CD147 and activates intracellular STAT3 signaling, we wondered whether the intracellular domain of CD147 is involved in signal transduction. We restored the expression of truncated CD147 (lacking the intracellular domain) in HCT-8 CD147KO cells and found that the intracellular domain of CD147 was dispensable for TFF3-induced STAT3 signaling activation and PTGS2 expression (Supplementary Fig. 10a, b).

It is plausible that CD147 may transduce the signal with the assistance of other molecules. CD147 can promote STAT3-mediated pancreatic cancer development by interacting with CD44s^18^ and we found that CD44s colocalized with CD147 in CRC cells (Supplementary Fig. 10c). Co-IP assays showed that CD147 formed a complex with CD44s and that TFF3 promoted the interaction between CD147 and CD44s (Fig. 4e). We also found that TFF3 interacted with CD147 but not CD44s (Supplementary Fig. 10d). Furthermore, FRET assays showed that TFF3 could increase the interaction between CD147 and CD44s on the cell membrane in live cells (Fig. 4f). CD44s knockdown led to decreased PTGS2 expression (Supplementary Fig. 10e), and gene expression analysis using GEO data showed that CD44s and PTGS2 expression levels were highly correlated (Supplementary Fig. 10f). Furthermore, silencing of CD44s eliminated the increase in PTGS2 expression induced by TFF3 (Fig. 4g).

To further elucidate CD44s-mediated downstream signaling induced by TFF3, an anti-CD44s antibody was employed to capture the signaling complex. CD44s formed a complex with SRC, and this interaction was enhanced by TFF3 (Fig. 4h). TFF3 promoted SRC activation in a time-dependent manner (Fig. 4i), and SRC inhibition by the SRC kinase inhibitor, KX2-391, could offset the effects of TFF3 on PTGS2 expression and STAT3 activation (Fig. 4j). These results indicate that the CD147–CD44s interaction is a key event in TFF3-induced SRC-mediated STAT3 activation and PTGS2 expression.

### The PGE2-PTGER4-cAMP-PKA-SRC pathway is a feed-forward loop that regulates STAT3 activation and PTGS2 upregulation in CRC cells exposed to TFF3

We found that TFF3 treatment increased intracellular cAMP levels (Supplementary Fig. 11a). The adenylate cyclase (AC) inhibitor MDL-12,330 A hydrochloride prevented TFF3-induced STAT3 phosphorylation (Supplementary Fig. 11b), PTGS2 expression (Supplementary Fig. 11c), SRC activation (Supplementary Fig. 11d), and PGE2 generation (Supplementary Fig. 11e). In contrast, the cAMP analog 8-bromo-cAMP increased STAT3 phosphorylation, SRC activation, and PTGS2 expression (Supplementary Fig. 11f) and PGE2 abundance (Supplementary Fig. 11g), and all these events could be blocked by the SRC inhibitor KX2-391 (Supplementary Fig. 11h). Consistently, PGE2 treatment enhanced STAT3 phosphorylation, SRC activation, and PTGS2 expression in a time-dependent manner (Supplementary Fig. 11i). Since PTGER4 was shown to be the main PGE2 receptor expressed on CRC cells (Supplementary Fig. 11j), we treated cells with the PTGER4 antagonist ONO-AE3, which significantly impaired TFF3-induced PTGS2 expression (Supplementary Fig. 11k) and PGE2-induced cAMP generation (Supplementary Fig. 11l), STAT3 phosphorylation and PTGS2 expression (Supplementary Fig. 11m).

These data indicate that TFF3 promotes PTGS2 expression and secondary PGE2 production and that PGE2, in turn, activates STAT3 and increases PTGS2 expression through PTGER4-cAMP-PKA-SRC signaling, suggesting a local feed-forward loop governing PTGS2 production in CRC cells exposed to TFF3.

### TFF3 promotes mucosal restitution via CD147-PTGS2-PGE2 signaling

**Fig. 5:**
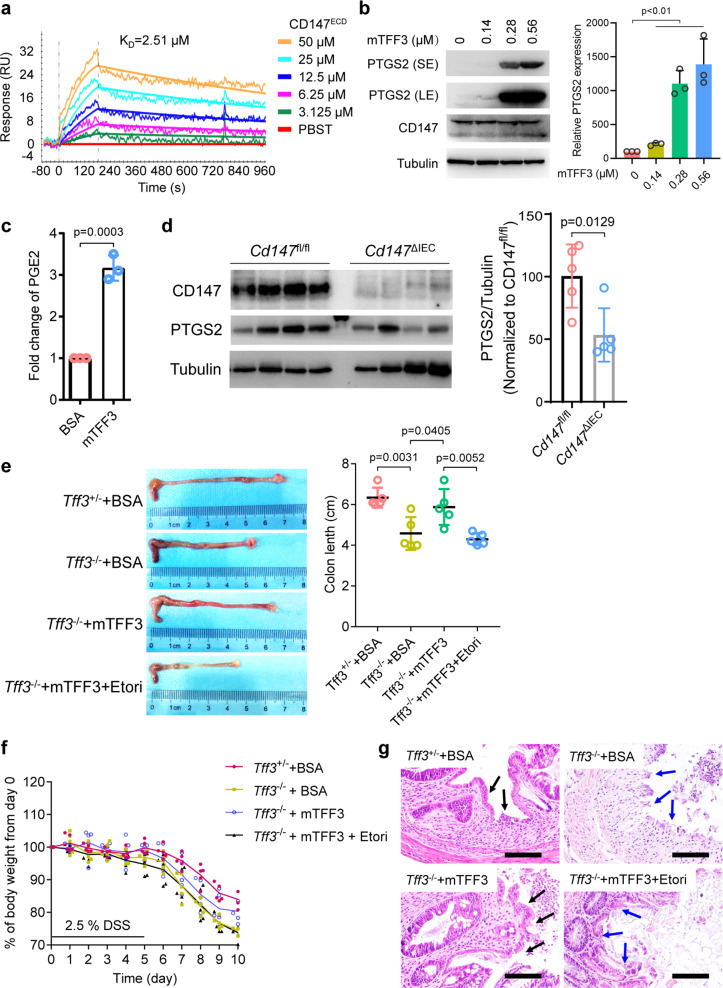
TFF3 promotes mucosal restitution via CD147-PTGS2-PGE2 signaling. **a** Biophysical analysis of the mTFF3-mCD147^ECD^ interaction using SPR. **b** Western blotting analysis of the indicated proteins in mouse primary IECs treated with increasing amounts of mTFF3. SE, short exposure, 20 s; LE, long exposure, 1 min. **c** Quantification of PGE2 in the culture supernatant of mouse primary IECs treated with 0.28 μM of BSA or mTFF3. **d** Western blotting analyses of the indicated proteins in colonic tissue of the floxed mice (*Cd147*^fl/fl^) or the intestinal epithelial-specific CD147 knockout mice (*Cd147*^ΔIEC^). **e** Representative macroscopic views of colons from mice of the indicated genotypes treated with BSA or mTFF3 (7.7 μM protein/20 g weight) alone or in combination with etoricoxib (Etori, 0.2 mg/20 g weight). The graph shows the average length of the colon. The *p*-values in **b**–**e** were determined by using a two-tailed Student’s *t*-test. **f** Weight loss (expressed as a percentage of the initial weight) over time (*n* = 5). **g** Representative areas of healing ulceration. Black arrow, healing, and re-epithelialization are apparent; blue arrow, healing is not apparent

Numerous in vivo and in vitro studies have indicated that TFF3 is a key player in mucosal protection and repair processes,^19^ and we investigated whether CD147-PTGS2-PGE2 signaling was responsible for TFF3-mediated mucosal restitution. We found that murine TFF3 (mTFF3) directly interacted with murine CD147^ECD^ (Fig. 5a). Furthermore, we isolated primary murine intestinal epithelial cells (IECs) and found that mTFF3 induced the expression of PTGS2 in a dose-dependent manner (Fig. 5b) and significantly increased PGE2 production (Fig. 5c). Conditional knockout of CD147 in IECs led to decreased PTGS2 expression in colonic tissues (Fig. 5d).

We also found that *Tff3*^−/−^ mice had a reduced repairability and lost more weight compared with *Tff3*^+/−^ mice (Fig. 5e–g), a similar phenotype was observed in a previous study.^20^ To confirm that this impairment of healing was secondary to the absence of TFF3 and the attenuation of PTGS2 signaling, we evaluated the ability of recombinant mTFF3 and etoricoxib to restore restitution. Rectal instillation of mTFF3 resulted in the reconstitution of normal healing with enhanced epithelial migration and attenuation of gross injury. In sharp contrast, the protective effect of mTFF3 was abolished by etoricoxib (Fig. 5e–g). These results suggest that TFF3 binds to CD147 and promotes mucosal restitution by inducing PTGS2.

### Inhibition of TFF3-CD147 signaling reduces metastasis of CRC cells to the lungs in vivo

**Fig. 6:**
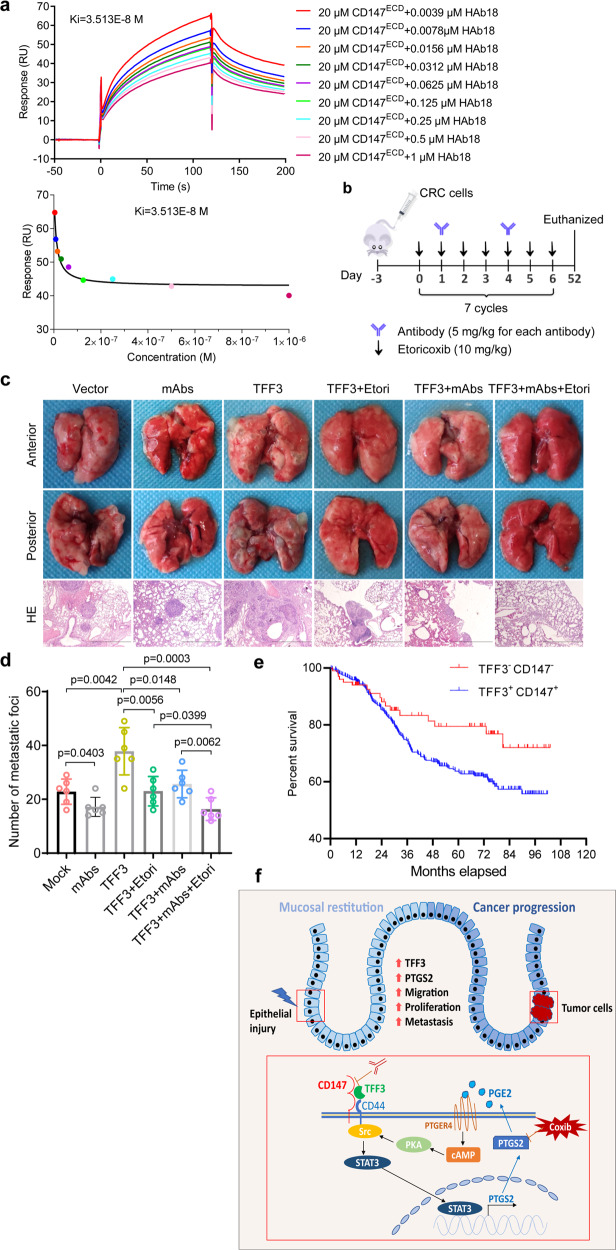
Blockage of TFF3-CD147 signaling reduces metastasis of CRC cells. **a** Biophysical analysis of the TFF3-CD147^ECD^ interaction in the presence of the monoclonal antibody HAb18 using SPR. The indicated concentrations of HAb18 and purified CD147^ECD^ were injected over immobilized TFF3. **b** Schematic representation of tail-vein injection of HCT-8–TFF3 or HCT-8-vector cells, as well as the schedule of PTGS2 inhibitor etoricoxib (Etori) and anti-CD147^ECD^ antibody administration in the nude mouse model of CRC metastasis. After injection of HCT-8-TFF3 cells, the mice were randomized to 4 treatment groups for 7 weeks: placebo, etoricoxib only, monoclonal antibodies only, and a combination of etoricoxib and monoclonal antibodies. After injection of HCT-8-vector cells, the mice were randomized to 2 groups for 7 weeks: placebo and monoclonal antibodies only (*n* = 6 in each group). **c** Representative images of lung metastases and HE staining of metastatic tumors that developed in the mouse model in **b**. Scale bar, 2 mm. **d** The lung metastases from each mouse were counted, and data are presented as a scatter diagram. The *p*-values were determined by using a two-tailed Student’s *t*-test. **e** Cumulative overall survival of patients with double positive or double negative tumor TFF3 and CD147 expression. **f** Model depicting the proposed mechanism mediating mucosal restitution and CRC progression

We have shown that TFF3-CD147 signaling promotes CRC cell migration, invasion, and proliferation, which are key events for distal metastasis. We then investigated the potential of targeting TFF3-CD147 signaling to prevent metastasis in vivo. Three in-house monoclonal antibodies (mAbs) (HAb18, 6H8, and 5A12) targeting different epitopes of CD147^ECD^ were used in competitive inhibition experiments using SPR. These experiments revealed that HAb18 and 6H8 could interfere with the interaction between TFF3 and CD147 (Fig. 6a, Supplementary Fig. 12). Thus, these two antibodies were used together in the subsequent experiments (Fig. 6b). We found that the selective PTGS2 inhibitor etoricoxib and the antibodies targeting CD147^ECD^ could weaken the ability of TFF3 to promote distal metastasis (Fig. 6c–d). Most importantly, mice treated with antibodies against CD147^ECT^ plus etoricoxib developed the fewest lung metastases, indicating that targeting TFF3-CD147 signaling might be a promising treatment strategy for preventing metastasis.

### Co-expression of TFF3 and CD147 correlates with CRC patient survival

To investigate whether the TFF3-CD147 ligand-receptor system is associated with pathological stage and patient survival, we performed an immunohistochemical analysis in 886 CRC tissues. Approximately 65.35% of the CRC specimens were positive for TFF3, whereas approximately 77.54% of them were positive for CD147 (Supplementary Table 9 and Supplementary Fig. 13). Notably, although the expression of TFF3 per se or CD147 per se did not correlate with the TNM stage, their co-expression did correlate with the TNM stage (Supplementary Table 9). Patients with a high expression of TFF3 and CD147 had lower survival compared to patients with low expression of TFF3 and CD147 (HR = 1.68, 95% confidence interval, 1.113–2.538, *p* = 0.0397; Fig. 6e). These results suggest that the TFF3-CD147 association might be used as a prognostic marker for CRC.

## Discussion

Numerous efforts have been made to characterize the TFF3 binding proteins; a range of potential TFF3-interacting proteins have been described. Using fresh CRC tissues, we identified CD147, a glycosylated protein with a molecular weight in the range of 33 to 66 KDa depending on the degree of glycosylation; moreover, CD147 was found to be critical for TFF3-induced cell migration, invasion, metastasis, and proliferation. Tan et al. found a 50 kDa glycosylated membrane protein, having the same size as that of CD147, as a potential binding partner of TFF3 in rat small intestine. In addition, we confirmed that mucin treatment could upregulate the membrane localization of CD147, consistent with the observation of Tan et al.^15^ Considering that TFF3 plays a wide variety of roles in various physiological and pathological conditions, it is not surprising that several TFF3 interacting partners, including membrane receptors for TFF3, may exist and that the interaction may be cell type-dependent and tissue context-dependent.

CD147 is a transmembrane protein in the immunoglobulin superfamily expressed in many cell types, including epithelial cells.^21^ Our findings are in agreement with studies showing that CD147 is expressed in normal tissues of the gastrointestinal tract and that a broad range of human malignant cancers, including CRC, have elevated CD147 expression.^22^ The results of this study show that CD147 is indispensable for TFF3-induced cell migration, invasion, and proliferation of CRC cells, and this is supported by previous findings regarding the roles of CD147 in inflammation.^23,24^ as well as promotion of proliferation, survival, angiogenesis, and the invasive properties of tumor cells.^25,26^

CD44s, an adhesion, and the antiapoptotic molecule are overexpressed in CRC and its expression is significantly associated with the depth of invasion and lymph node involvement.^27^ Our previous data suggest that CD44s are associated and co-localized with CD147 in pancreatic cancer.^18^ Here, we show that TFF3 binds to CD147 and enhances the interaction between CD147 and CD44s, resulting in SRC and STAT3 activation. Based on proteomics analysis, we found that PTGS2 was a downstream effector responsible for TFF3-induced cell migration, invasion, and proliferation. TFF3 induced PTGS2 expression mainly by activating STAT3 signaling (Fig. 6f). Prolonged elevation of PTGS2 expression appears to contribute significantly to long-term changes in intestinal tract function and cell proliferation.^28^ Elevated PTGS2 levels have been previously reported to promote carcinogenesis, tumor proliferation, infiltration, metastasis, angiogenesis, and tumor resistance to anti-cancer drugs.^29^ Activated STAT3 is increased in intestinal epithelial cells during active inflammatory bowel disease (IBD), ulcerative colitis (UC)-associated high-grade dysplasia, and cancer.^30^ Mice with conditional knockout of STAT3 in intestinal epithelial cells have been found to be highly susceptible to experimental colitis, indicating that epithelial STAT3 regulates intestinal homeostasis.^31^ Epithelial STAT3 is essential for mucosal wound healing by promoting regeneration of the epithelium in response to injury, thereby mediating recovery from colitis.^32^ In addition, levels of activated STAT3 are known to be remarkably elevated in patients with CRC and the high activated STAT3 levels correlate with tumor invasion, metastasis, and worse prognosis.^33,34^

TFF3 and PTGS2 have been reported to play pivotal roles in the maintenance and repair of the intestinal mucosa. Mashimo et al. showed that mice lacking TFF3 had impaired mucosal healing, with poor epithelial regeneration after injury^20^ and the same was observed following chemotherapy and radiation-induced damage.^35^ Here, we provide another piece of evidence that *Tff3*^−/−^ mice exposed to DSS to provoke acute colonic inflammation showed more severe tissue damage, more loss of body weight, and worse tissue repair than *Tff3*^+/−^ mice. A recent study reported an inconsistent observation that *Tff3*^*−/−*^ mice were not more susceptible to DSS-induced colitis than wild-type mice.^14^ There could be several reasons for this discrepancy, including intestinal microbiota and dosage regimen. In addition, we showed that TFF3 induced PTGS2 expression by binding to CD147 in human and mouse colonic epithelial cells. The protective effect of TFF3 was abolished by a PTGS2 inhibitor, indicating that TFF3 functions in mucosal defense against acute colonic injury mainly through PTGS2. PGE2 is synthesized by PTGS2 and functions as a crucial inflammatory cytokine. PGE2 is the most abundant prostanoid found in CRC tissue, and it has been shown to play an important role in cellular regeneration and CRC progression.^36,37^ We demonstrated that TFF3 promotes PTGS2 and PGE2 expression. Given that TFF3 expression is increased in the tumor tissues and sera of patients with CRC, and that the TFF3 receptor CD147 expression is also increased in colon cancer,^22^ we suggest that elevated TFF3 binds to upregulated CD147 and promotes CRC progression by inducing PTGS2 expression and PGE2 production. Consistent with previous findings,^38,39^ we found that PTGS2 itself could be induced by PGE2 treatment. Therefore, it is appropriate to conclude that a feedforward loop exists in cells exposed to TFF3. We speculate that this positive feedback loop enables TFF3 to induce quick signal amplification after tissue injury and promote CRC progression at relatively low concentrations.

Animal experiments, epidemiological studies, and clinical trials indicate that nonsteroidal anti-inflammatory drugs (NSAIDs) and selective PTGS2 inhibitors (coxibs) are among the most promising chemopreventive and therapeutic agents for cancer,^40–42^ however, selective PTGS2 agents have been found to be associated with an increased risk of cardiovascular side effects.^43^ Although oncogenic SRC family kinases are potent drivers of CRC metastasis, clinical trials using small SRC inhibitors in CRC failed.^44^ STAT3 has been well validated as an attractive anticancer target due to its important roles in cancer initiation and progression, while there has been no STAT3 targeted drug approved for clinical application.^45^ In our study, we showed that inhibition of TFF3-CD147 signaling using competitive inhibitory antibodies reduced CRC lung metastasis. Neutralizing elevated TFF3 to reduce SRC/STAT3 activation and PTGS2 induction or blocking the interaction between TFF3 and its receptor, CD147, may be a promising strategy for the chemoprevention and treatment of CRC.

Our findings not only identified CD147 as a novel receptor for TFF3 but also uncovered a novel mechanism for the contribution of the TFF3-CD147 ligand-receptor system to mucosal restitution and CRC progression. Moreover, our in vitro and in vivo results may provide a rationale for the development of new therapeutic agents targeting the TFF3-CD147 interaction interface for the prevention and treatment of gastrointestinal disorders.

## Materials and methods

### Primary human CRC cells

Two fresh colon cancer tissues were collected from the Department of Gastrointestinal Surgery, Xijing Hospital, which is affiliated with the Fourth Military Medical University. Both individuals were male and provided written informed consent, and the study was approved by the Hospital Ethics Committee (KY20163269-2). Human CRC specimens were cut into small (approximately 1 mm^3^) pieces, which were then washed three times with DMEM. The pieces were digested in DMEM supplemented with 2 mg/mL collagenase III (Worthington Biochemical Corp.) at 37 °C and with 5% CO_2_ for 5 h with occasional shaking. Human epithelial tumor cells were isolated using human EpCAM MicroBeads (Miltenyi Biotec) according to the manufacturer’s instructions. Isolated cells were cultured in DMEM supplemented with 10% FBS and 1% penicillin-streptomycin.

### Cell lines

The HCT-8, HCT116, SW480, SW1116, HEK293, and FHC cell lines were purchased from American Type Culture Collection and cultured in RPMI-1640 medium supplemented with 10% fetal bovine serum (FBS). The CD147-knockout cell line HCT-8 CD147KO was generated using the CRISPR/Cas9 system. All cells were cultured at 37 °C and with 5% CO_2_.

### Mice

All animal protocols were approved by the Animal Care and Welfare Committee of the Fourth Military Medical University. Mice with a C57BL/6 background weighing ~20 g were used in this study. *Tff3*^−/−^ mice were generated as described previously by Biocytogen.^20^ *Cd147*^f/f^ mice were established in our laboratory.^46^ *Villin*^Cre/+^ mice were obtained from Jackson Laboratory. The PCR primers used for the genotyping of mice are listed in Supplementary Table 10. Immunodeficient nude mice (BALB/c, 6–8 weeks old) were obtained from Beijing HFK Bioscience.

### DSS-induced colitis

Male mice weighing ~20 g were used in this study. A 2.5% solution of DSS in drinking water was administered for the first 5 days, followed by regular drinking water for another 5 days. The mice were monitored daily for their body weight. On day 10, mice were euthanized and their colon lengths were measured.

### Xenograft tumor model

In total, 2 × 10^6^ cells in 0.1 mL RPMI 1640 medium were subcutaneously inoculated into the right posterior flank of nude mice. After 20 days, the mice were euthanized by cervical dislocation, and the tumor tissues were analyzed by hematoxylin and eosin (HE) and immunohistochemical staining.

### Metastasis assays

Nude mice were injected with 2 × 10^6^ cells in 0.1 mL saline solution via the lateral tail vein. After 52 days, the mice were euthanized, and the lungs were removed immediately and photographed to count pulmonary metastatic nodules in each of the lobes.

### Mass spectrometry analysis

An LTQ-Orbitrap hybrid mass spectrometer (Thermo Fisher Scientific) was used to obtain tandem mass spectra of tryptic peptides. The resulting tandem mass spectrometry spectra were analyzed using the SEQUEST algorithm against a nonredundant human protein database (NCBI, Feb 2007) for putative protein identification. The iTRAQ experiment was carried out on a Triple TOF 5600 system (AB Sciex) coupled with a Nanoflex microchip system (Eksigent) as previously described.^47^

### Co-IP analysis

Co-IP analysis was performed using a co-IP kit (Pierce) according to the manufacturer’s instructions. Briefly, 15 μg of affinity-purified antibody was coupled to the resin. The antibody-agarose beads were mixed with the lysate, and the mixture was incubated with gentle mixing at 4 °C overnight. After washing with ice-cold IP lysis/wash buffer six times, the immunoprecipitated proteins were then eluted and detected via western blotting.

### Western blotting

Equal amounts of protein were denatured in 5× Laemmli buffer (Beyotime), separated via 10%, 12%, or 15% SDS-PAGE, and transferred onto PVDF membranes (Millipore). The membranes were blocked with 5% non-fat milk in Tris-buffered saline containing 0.1% Tween-20 (TBST) and then incubated with primary and secondary antibodies according to the manufacturer’s instructions. Immunoblots were developed using an ECL kit (Beyotime).

### Quantitative real-time PCR analysis

Total RNA was extracted using TRIzol reagent (OMEGA Bio-Tek). Reverse transcription was performed using the PrimeScript RT reagent kit (TaKaRa Biotechnology). All primers were synthesized by BGI. Primer sequences are listed in Supplementary Table 10. Real-time PCR was performed using the SYBR Premix Ex Taq II Kit (TaKaRa Biotechnology) on an Agilent Mx3005P, and data were analyzed with MxPro-Mx3005P software.

### In vitro wound-healing assay

A pipette was used to scratch the monolayer. The cells were then washed with a serum-free medium. Photomicrographs were obtained at various time points (0 h and 24 h), and the migration distance was analyzed using Image J. The relative migration distance was calculated using the following formula: relative migration distance (%) = 100 (A treatment − B treatment)/(A blank – B blank), where A is the width of the cell wound before incubation, and B is the width of the cell wound after incubation.

### In vitro invasion assay

This assay was performed using chambers containing polycarbonate filters (8 μm pore size, Millipore). The upper side of a polycarbonate filter was coated with matrigel (BD Bioscience, San Jose, CA). CRC cells (1 × 10^5^) were added to the upper chamber. The lower chamber was filled with a 10% FBS-containing medium. After a 24 h incubation, the cells were fixed and stained with 0.2% crystal violet in 95% ethanol at room temperature for 30 min. The chambers were washed with PBS several times to remove excess dye. The cells on the underside were counted.

### CCK-8 assay

CCK-8 solution (10 μL) at a 1:10 dilution with serum-free RPMI 1640 (100 μL) was added to each well, followed by a further 2 h incubation. The absorbance was automatically measured at 450 nm with a microplate reader (Epoch, BioTek Instruments).

### Apoptosis and cell cycle assays

Apoptosis and cell cycle rates were assessed using an Annexin V-FITC/propidium iodide (PI) apoptosis detection kit and a cell cycle detection kit, respectively (KeyGEN Biotech). Quantification of PI and FITC signals was performed using a fluorescence-activated cell sorter FACSAria system (BD Bioscience).

### Mucin treatment assay

Mucin (from pig stomach) was supplied by Sigma Chemical Co. HCT-8 cells were cultured in a medium containing various concentrations of mucin at 37 °C for 90 min.

### cAMP assay

The cAMP assay was performed using a human cAMP ELISA kit (Shanghai Enzyme-linked Biotechnology) according to the manufacturer’s instructions.

### Immunofluorescence staining

After being washed twice with PBS, the cells were fixed in paraformaldehyde in PBS, permeabilized with 0.1% Triton X-100, and blocked with 1% bovine serum albumin (BSA) in PBS for 1 h. The cells were then incubated with the appropriate primary antibodies and secondary antibodies. Cell nuclei were stained with DAPI (Vector Labs). The cells were visualized using an A1R-A1 confocal laser microscope system (Nikon).

### FRET assay

Three sequential images were acquired in the same field with suitable filter sets for the donor (EGFP; excitation at 488 nm and emission at 515 nm), acceptor (DsRed; excitation at 543 nm and emission at 585 nm), and FRET (excitation at 488 nm and emission at 585 nm). The correction coefficients for each of the fluorescent proteins were calculated using the following equations and variables. CoB = (Dad)/(Ddd), where Dad is the average intensity of the image acquired from the donor-only sample using the FRET filter setup, and Ddd is the average intensity of the image acquired from the donor-only sample by using the donor filter setup. CoA = (Daa)/(Aaa), where Daa is the average intensity of the image acquired from the acceptor-only sample by using the FRET filter setup, and Aaa is the average intensity of the image acquired from the acceptor-only sample by using the acceptor filter setup. FRET calibration and net FRET were calculated using analysis software (Nikon).

### Immunohistochemistry (IHC)

IHC was performed as previously described.^48^ The expression levels were independently evaluated by two senior pathologists according to the proportion and intensity of positive cells. The following criteria were used to score each specimen: 0 (no staining), 1 (any percentage with weak intensity or <30% with strong intensity), 2 (30–50% with strong intensity), and 3 (>50% with strong intensity).

### Protein preparation

The extracellular portion of CD147 was prepared as previously described.^49^ For ^15^N isotopic labeling, the bacteria were grown in a ^15^N-labeled M9 minimal medium. The DNA sequence encoding a mature form of TFF3 (residues 22-80) was subcloned into a modified pBAD33-ssMBP vector (gifted by Dr. Shu Quan lab at East China University of Science and Technology). For His_6_-tagged TFF3, a His_6_ tag was inserted to the C-terminus of TFF3. The protein was purified with an MBPTrap HP column (GE Healthcare). TFF1 was purchased from Sino Biological. TFF2 was obtained from Novoprotein.

### NMR titration experiment

The NMR samples all contained 0.1 mM uniformly ^15^N-labeled protein in 50 mM Tris-HCl and 50 mM NaCl (pH 7.5) with 90% H_2_O/10% D_2_O. A series of two-dimensional ^1^H–^15^N HSQC spectra with gradually increased TFF3 protein concentrations (0.1 mM, 0.2 mM, and 0.4 mM) were collected at 298 K on a Bruker Avance 900 MHz spectrometer with 4-mm NMR tubes equipped with a triple-resonance cryoprobe. All NMR spectra were processed with NMRPipe and analyzed using SPARKY. ^1^H and ^15^N backbone amide chemical shifts, Δ*δ*(N–H), were computed as:

$${\Delta}\delta \left( {{\mathrm{N}} - {\mathrm{H}}} \right) = \sqrt {\left( {{\Delta}{\mathrm{H}}\delta {\mathrm{ppm}}} \right)^2 \,+\,\,\left( {\frac{{{\Delta}{\mathrm{N}}\delta {\mathrm{ppm}}}}{5}} \right)^2}$$

### Serum collection and ELISA

Whole blood was collected from the Department of Gastrointestinal Surgery, Xijing Hospital, and processed to collect the serum. All individuals provided written informed consent, and the study was approved by the Hospital Ethics Committee (KY20163269-1). TFF3 ELISAs were performed according to the manufacturer’s instructions (BOSTER).

### SPR

SPR studies were performed using a Biacore T200 instrument. TFF peptides were diluted in 10 mM sodium acetate (pH 4.0) and immobilized on a CM5 chip (Biacore, GE Healthcare). Purified proteins (as analytes) were injected over the chip at 30 µL/min with a 2 min association time and varied dissociation times. Multiple cycle kinetics were fit using a mathematical model of a simple 1:1 interaction in the Biacore T200 evaluation software (Biacore).

### Statistics

Quantitative results are presented as the mean ± SD and individual data points are plotted. Statistical analyses are described for each panel. Differences were compared by using a two-tailed Student’s *t*-test as indicated in the figure legends. Spearman’s R was used to determine correlations between the relative expression of genes. All reported *p*-values were two-tailed, and *p* < 0.05 was considered significant.

## Supplementary information

Supplementary information

## Data Availability

The data that support the findings of this study are within the Article, Supplementary Information, or available from the corresponding author upon reasonable request.
