# Supplementary material for: CD147 receptor is essential for TFF3-mediated signaling regulating colorectal cancer progression
Source: Signal Transduct Target Ther. 2021 Jul 14;6:268. doi: 10.1038/s41392-021-00677-2 (PMC8280106; doi:10.1038/s41392-021-00677-2)
Supplement: Supplementary file 1 — Supplementary information [file 41392_2021_677_MOESM1_ESM.docx]

Supplementary Materials for

CD147 receptor is essential for TFF3-mediated signaling regulating colorectal cancer progression

Hong-Yong Cui, Shi-Jie Wang, Fei Song, Xu Cheng, Gang Nan, Yu Zhao, Mei-Rui Qian, Xi Chen, Jia-Yue Li, Fen-Ling Liu, Yu-Meng Zhu, Ruo-Fei Tian, Bin Wang, Bin Wu, Yang Zhang, Xiu-Xuan Sun, Ting Guo, Xiang-Min Yang, Hai Zhang, Ling Li, Jing Xu, Hui-Jie Bian, Jian-Li Jiang, and Zhi-Nan Chen

Correspondence to: [znchen@fmmu.edu.cn](mailto:znchen@fmmu.edu.cn) (Z.N.C.), [jiangjl@fmmu.edu.cn](mailto:jiangjl@fmmu.edu.cn) (J.L.J.) and [hjbian@fmmu.edu.cn](mailto:hjbian@fmmu.edu.cn) (H.J.B.).

**This PDF file includes:**

Materials and Methods

Figures S1 to S13

Tables S1 to S10

**Materials and Methods**

**ChIP assay**

The binding of STAT3 to *PTGS2* promoter after treatment with or without TFF3 (0.152 μM) for 24 hours was measured with ChIP assay. Upon termination of treatment, HCT-8 cells were fixed with 1% formaldehyde, which was terminated with 1 M glycine. After washing with ice-cold PBS, the cells were scraped off and lysed with 1% SDS lysis buffer supplemented with protease inhibitor cocktail on ice. The lysed cells were sonicated to shear the chromatin DNA to an optimal size about 200~1000 bp. Sheared chromatin DNA was immunoprecipitated with antibodies against STAT3 (Cell Signaling Technology). Equal amounts of IgG served as negative control. The immunoprecipitate was then incubated with Magnetic Protein A/G Beads (Millipore) and pulled down on magnetic stand. After washing, reverse cross-linking was performed in 5 M NaCl at 65°C overnight. Contaminating RNA was cleaned with ribonuclease A, and protein was digested with proteinase K. Finally, the sheared DNA recovered from reverse cross-linking was extracted using DNA extraction kit for further quantitative analysis with qRT-PCR. The same amount of sheared DNA without antibody precipitation after reverse cross-linking served as input control. The ratio of DNA precipitated by STAT3 antibody over input control was obtained to indicate the amounts of bound transcription factor.

**Primary mouse IECs**

The intestines of newborn C57BL/6 mice were isolated and cut into small (approximately 1 mm^3^) pieces, that were then washed three times with Dulbecco’s modified Eagle’s medium (DMEM) in a culture dish. The pieces were digested with 1 mL of 1 mg/mL thermolysin for 30 min at 37°C with repeated pipetting and then filtered through a 40 μm filter. The crypts were retained on the filter, washed several times to remove fibroblasts, resuspended in DMEM supplemented with 10% FBS and penicillin-streptomycin and cultured at 37 °C with 5% CO_2_ and 95% humidity.

**Bioinformatics analysis**

RNA sequence data for 163 samples from patients with CRC and 10 normal intestinal mucosa samples were retrieved from TCGA. RNA sequence data for 507 normal intestinal mucosa samples were retrieved from the GTEx data portal. Before conducting differential expression analysis, all unexpressed RNAs with a mean read of one or less were filtered out. Differential expression analysis was performed using the DESeq2 R package (1.10.1). The resulting p values were adjusted using Benjamini and Hochberg’s approach for controlling the FDR. Genes with an adjusted p-value <0.05 and |log2 (fold change)| >1 found by DESeq2 were categorized as differentially expressed. Overall survival analysis was performed using the R survival package. The Kaplan-Meier method was used to fit the Kaplan-Meier curves. The log-rank test was used to compare the survival curves of the two groups.

**Generation of mAbs against CD147**

Three in-house mAbs against CD147 (HAb18, 6H8 and 5A12) were generated using the traditional hybridoma technology. Briefly, single-cell suspensions were prepared from hepatocellular carcinoma (HCC) surgical specimens, and BALB/c mice were immunized with an intraperitoneal injection of 1 × 10^7^ HCC cells. After three rounds of immunization, B cells from the spleen of the immunized BALB/c mice were fused with Sp2/0 cells to generate hybridoma cell clones. All the supernatants of the cell clones were screened via immunohistochemical staining of HCC tissue slices and cell-based ELISA to determine the specificity of the obtained mAbs. One of the positive cell clones was named HAb18 or metuximab. After screening the human HCC cDNA library with mAb HAb18 to identify the corresponding antigen, a cDNA sequence identical to the open reading frame of human CD147 (EMMPRIN, Basigin) was obtained. The isotype of HAb18 is mouse IgG1. For 6H8 and 5A12, BALB/c mice were immunized with purified CD147 protein, but not HCC cells. Two of the positive hybridoma clones were named 6H8 and 5A12, respectively. The specificity of the three mAbs was validated in our previous studies using CD147KO and CD147KD cells.

**Receptor screening**

Fresh colon cancer tissues were collected from the Department of Gastrointestinal Surgery, Xijing Hospital, which is affiliated with the Fourth Military Medical University. All individuals provided written informed consent, and the study was approved by the Hospital Ethics Committee (KY20163269-1). The precooled tissues were ground using TissueLyser II (QIAGEN) according to the manufacturer’s protocol and then lysed with Pierce Lysis buffer (Thermo Fisher Scientific). A His pull-down assay was performed using Pierce™ His Protein Interaction Pull-Down kit (Thermo Fisher Scientific) according to the manufacturer’s instructions, where His_6_-tagged TFF3 proteins were the bait, and the lysates from the fresh CRC tissues described above were the prey. The eluted proteins were identified via mass spectrometry.

**Electrophoretic mobility shift assay (EMSA)**

EMSA experiments between STAT3 and probes with biotin label at 5′-end were carried out using a Chemiluminescent EMSA Kit (Beyotime). The samples containing 0.1 pM probes and different contents of nuclear extracts were premixed and incubated for 20 min at room temperature, and subsequently loaded into a 4% native PAGE gel in 0.5× TBE buffer. After separation by electrophoresis, the free probe and the complex of probe and protein were transferred onto a nylon membrane and subjected to UV crosslinking. Subsequently detection of chemiluminiscence was conducted following the protocol of the Chemiluminescent EMSA Kit.

**Figure. S1.**


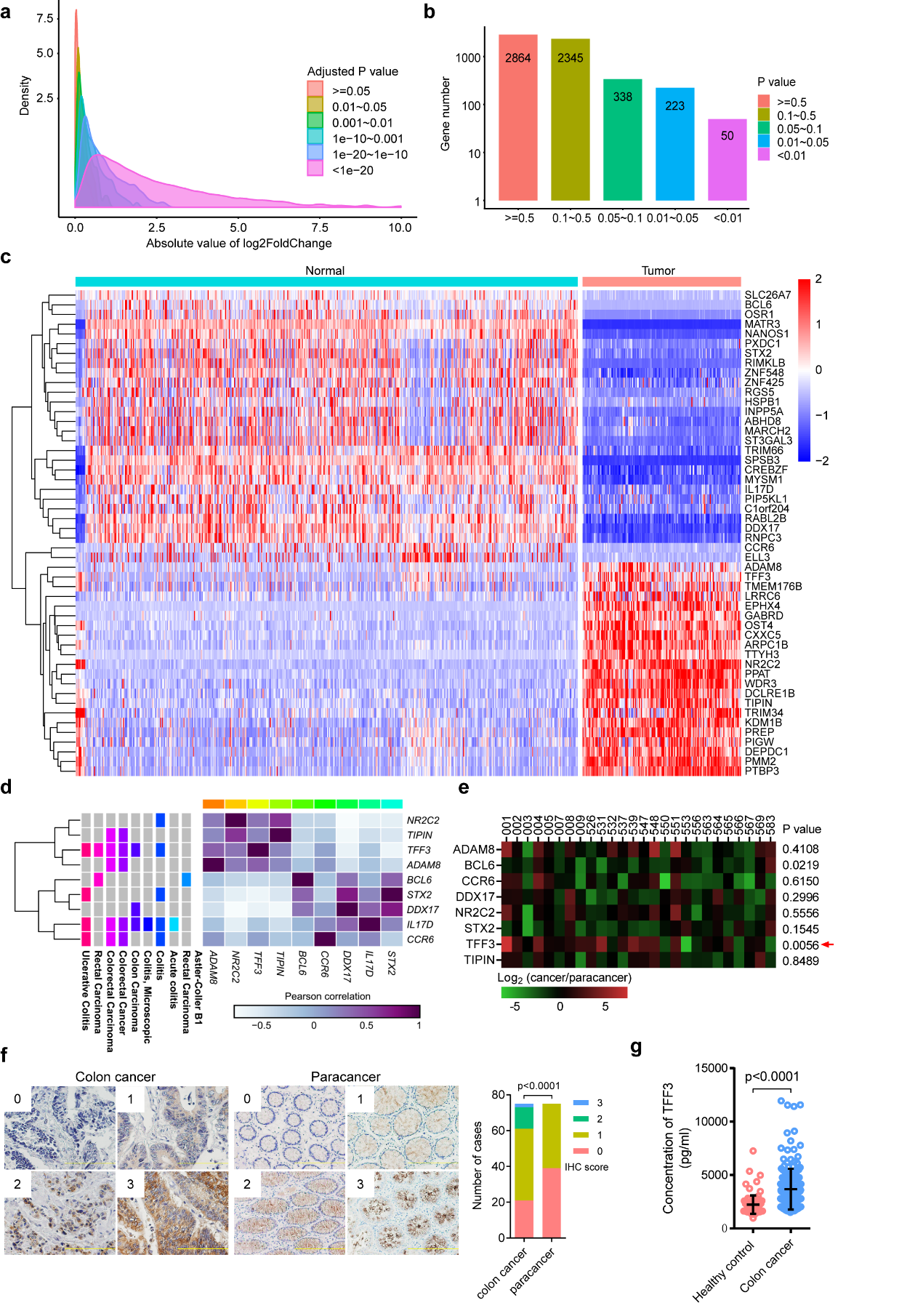
**Supplementary Fig. 1 Augmented TFF3 expression in CRC. a** Density distribution of DEGs between 163 colorectal cancer tissues and 517 normal mucosa grouped by adjusted p-value. The log2-transformed expression level and adjusted p-value of each gene were calculated by DESeq2 R package (1.10.1). **b** Distribution of DEGs identified in (**a**) with adjusted p < 1e-20 (n = 5,820). Genes were grouped by p-value derived from the overall survival analysis. **c** Heatmap of DEGs correlating with overall survival in CRC patients with a p < 0.01. **d** Heatmap of genes associated with colorectal disease using the DisGeNET database. **e** Heatmap of the selected genes in 26 CRC tissues and paired paracancer tissues. **f** Representative images of immunohistochemical staining of TFF3 in colon cancer tissues (left panel) and paracancer tissues (middle panel). The numbers represent IHC scores. Scale bar, 200 μm. Quantitation of TFF3 staining intensity in 75 CRC and paired normal mucosa (χ^2^=19.611, right panel). The p-value was determined by using Fisher’s exact test. **g** Serum TFF3 ELISA in CRC patients and healthy controls. The p-value was determined by two-tailed Student’s t-test.

**Figure. S2.**


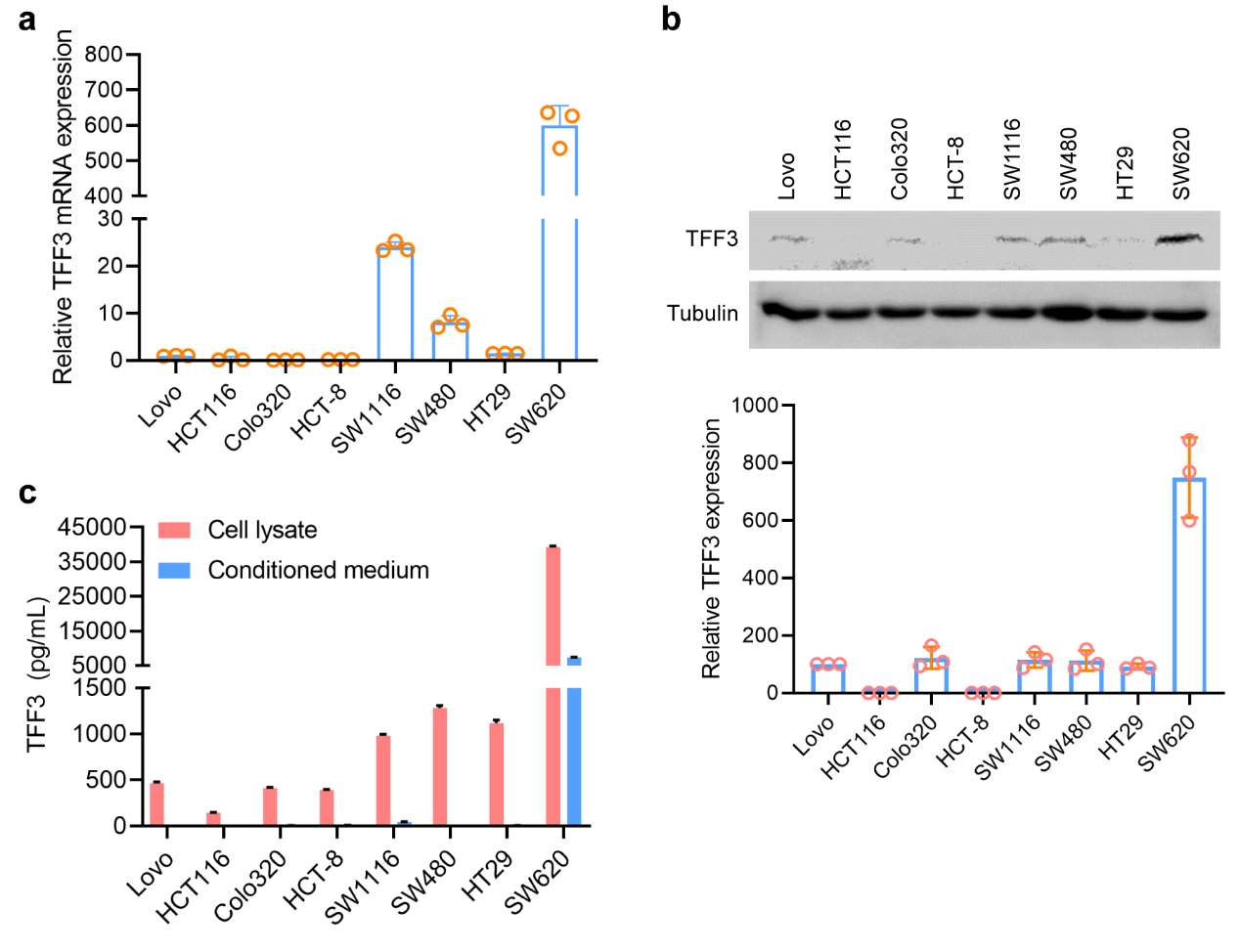


**Supplementary Fig. 2 Expression and secretion of TFF3 in CRC cell lines. a** *TFF3* mRNA expression analysis in colorectal cancer cell lines. The *TFF3* mRNA expression level in Lovo cells is set to 1. **b** Western blotting analysis of TFF3 expression in CRC cell lines. Graph shows semi-quantitative analysis of relative TFF3 expression. **c** TFF3 concentration in cell lysate and conditioned medium of CRC cell lines.

**Figure. S3.**

**
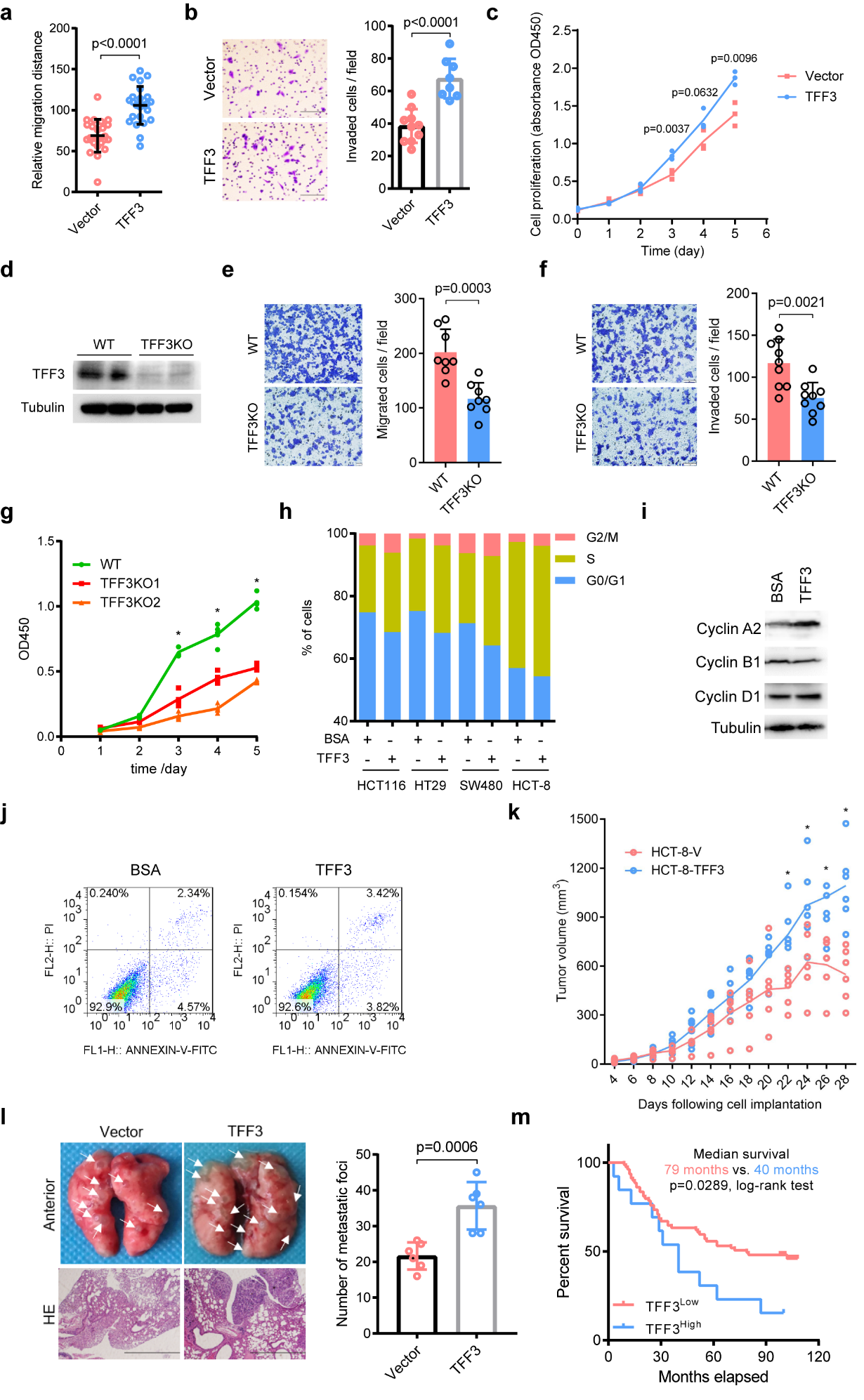
**

**Supplementary Fig. 3 TFF3 promotes cancer progression and correlates with poor survival in CRC. a** Quantification of cell migration ability of HCT-8 cells overexpressing TFF3 or control vector using wound healing assays. **b** Representative images of HCT-8 cells overexpressing TFF3 or control vector invading through matrigel-coated transwell inserts towards serum for 24 h. Scale bar, 50 μm. The graph shows the average number of invaded cells per field. **c** Proliferation curves of HCT-8 cells overexpressing TFF3 or control vector determined by CCK-8 assay. **d** Western blotting analysis of TFF3 expression in SW620 cells. **e** Representative images of SW620 cells invading through transwell inserts towards serum for 24 h. Scale bar, 50 μm. The graph shows the average number of invaded cells per field. **f** Representative images of SW620 cells invading through matrigel-coated transwell inserts towards serum for 24 h. Scale bar, 50 μm. The graph shows the average number of invaded cells per field. **g** Proliferation curve of SW620 cells determined by CCK-8 assay. TFFKO1 and TFF3KO2 were two independent TFF3 knockout clones. Significance relative to WT was determined by using two-tailed Student’s t-test (*p < 0.05). **h** Cell cycle distribution of indicated cells treated with BSA or TFF3. **i** Western blotting analyses of indicated proteins in HCT-8 cells treated with BSA or TFF3. **j** Analysis of apoptosis by flow cytometry. **k** Tumor volume of nude mice with subcutaneous colorectal cancer xenografts. n=6. **l** Representative images of lung metastases and HE staining in metastatic tumors. Scale bar, 2mm. The lung metastases from each mouse were counted, and data are presented as a scatter diagram.The p-values in (**a-c**, **e-g**, **k-l**) were determined by using two-tailed Student’s t-test. **m** Cumulative overall survival of patients with high (IHC score≥2) and low (IHC score≤1) tumor TFF3 expression. The p-value was determined using log-rank test.

**Figure. S4.**


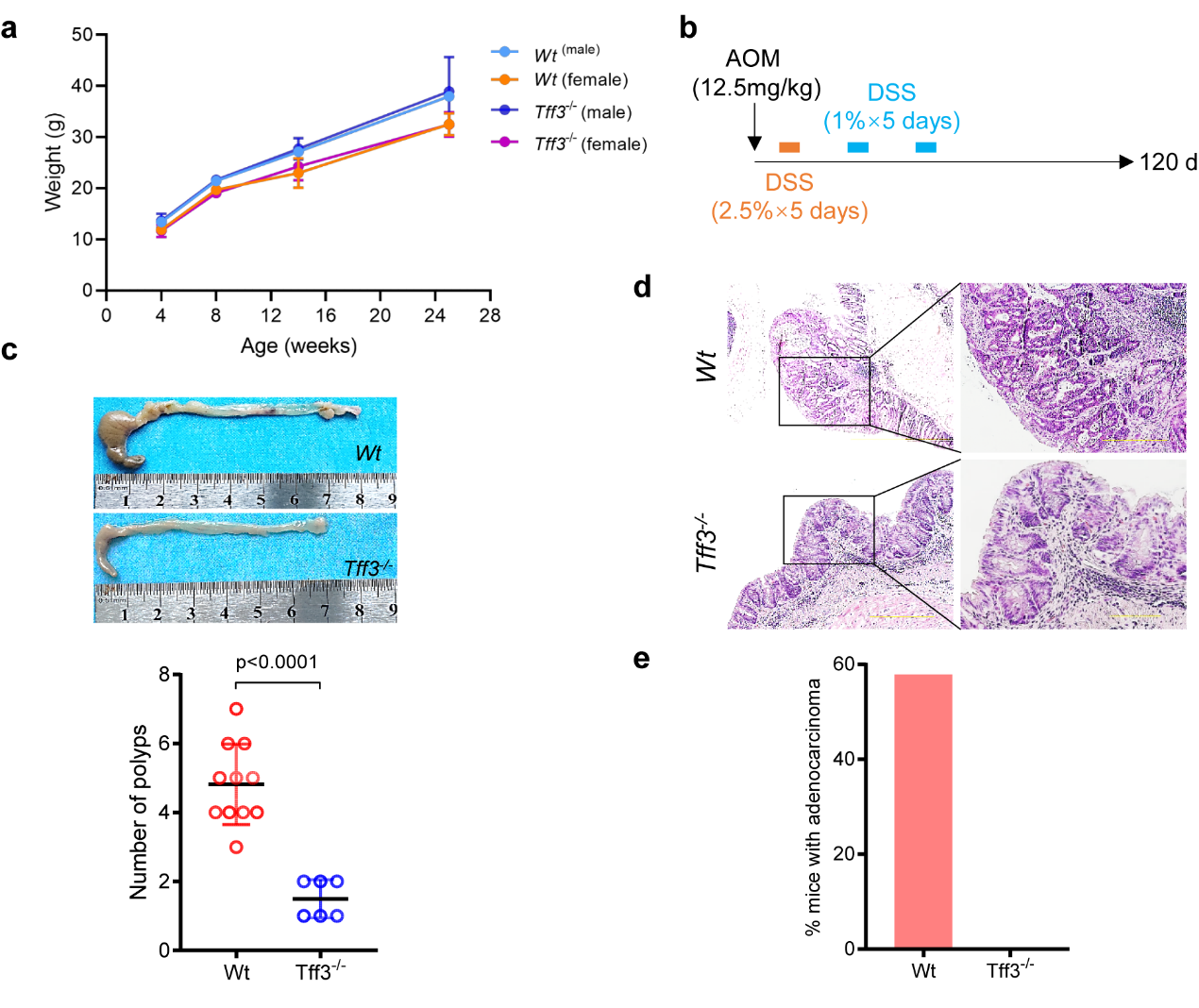


**Supplementary Fig. 4 Loss of *Tff3* decreases frequency of colonic adenocarcinoma development. a** Body weight of Wt and *Tff3^-/-^* mice. **b** Cohorts of 35 *Tff3^-/-^* mice and 36 *Wt* mice were injected i.v. with AOM on day 0 followed by three DSS cycles. **c** Representative macroscopic view of colons from mice of indicated genotypes. The polyps of all the colons were analyzed at the completion of the experiment, and data are presented as a scatter diagram. The p value was determined by using Student’s t-test. **d** Photomicrographs of representative sections from the respective groups are shown. **e** The percentage of mice developing adenocarcinomas is indicated.

**Figure. S5.**


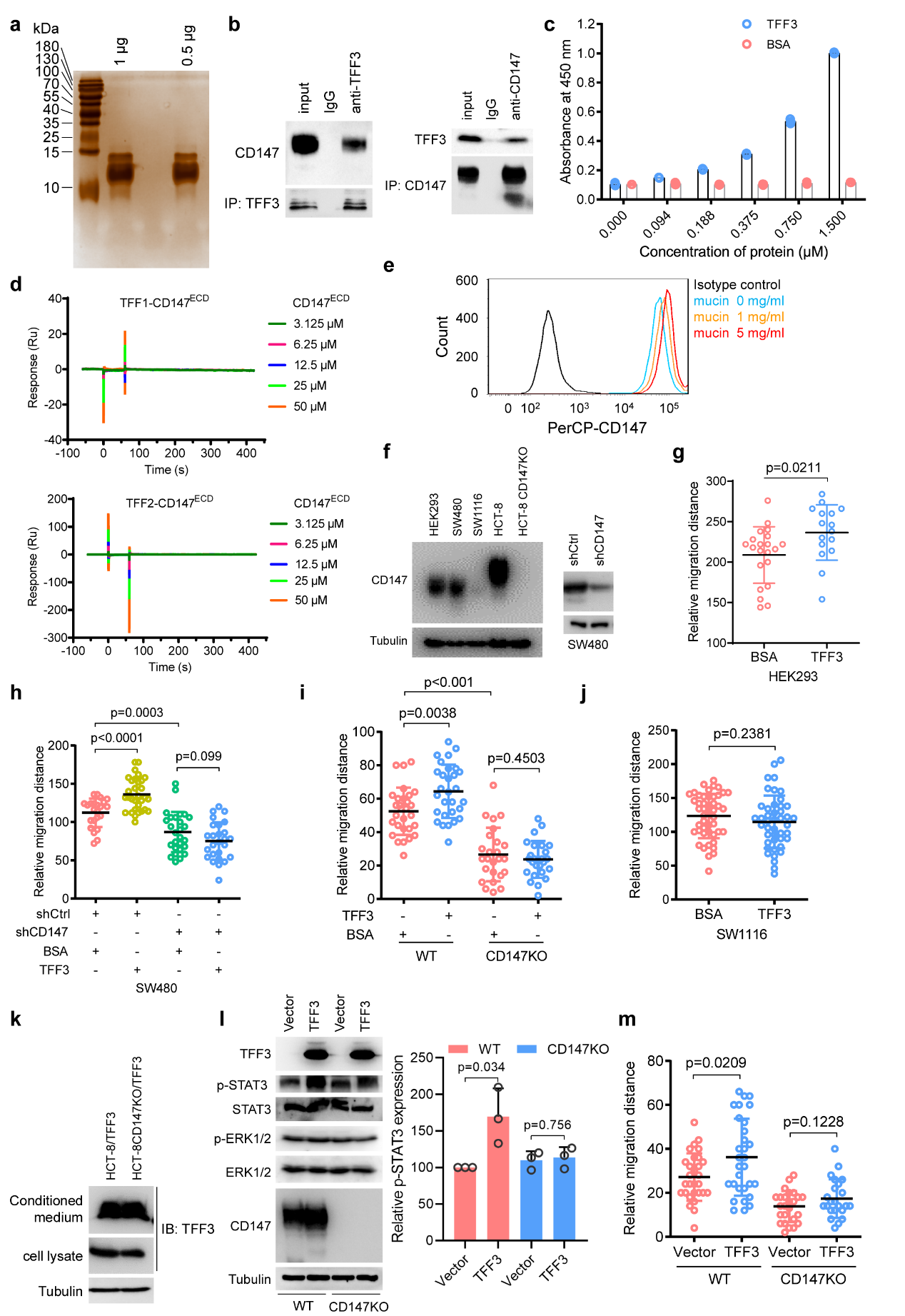


**Supplementary Fig. 5 CD147 is identified as a TFF3-binding protein and is indispensable for promoting migration and activating downstream signaling by TFF3. a** Silver staining of purified TFF3 protein. **b** Western blotting analyses of endogenous TFF3 co-IP with endogenous CD147 in SW620 cells. IgG was used as a control antibody. **c** ELISA for detecting the interaction between immobilized CD147^ECD^ and human TFF3 at various concentrations. BSA is shown as a negative control. **d** Biacore analysis of interaction between TFF1, TFF2 and CD147^ECD^. **e** Flow cytometry analysis of CD147 in HCT-8 cells treated with the indicated concentrations of mucin. **f** Western blotting analyses of CD147 expression in a panel of CRC cells. shCD147, shRNA targeting CD147. shCtrl, control shRNA. **g-j** Quantification of cell migration ability of HEK293 (**g**), SW480 (**h**), HCT-8 (**i**) and SW1116 (**j**) cells using wound healing assays. Cells were treated with 0.152 μM TFF3. **k** Western blotting analysis of TFF3 in cell lysate and conditioned medium of HCT-8 and HCT-8 CD147KO cells transfected with TFF3. 5% of cell lysate or 0.83% of conditioned medium from the same well were loaded onto the gel. **l** Western blotting analyses of the indicated proteins in HCT-8 or HCT-8 CD147KO cells transfected with control vector or TFF3. Graph shows semi-quantitative analysis of relative p-STAT3 expression. **m** Quantification of cell migration ability of HCT-8 and HCT-8 CD147KO cells transfected with control vector or TFF3 using wound healing assays. The p-values in (**g**-**j**, **l**-**m**) were determined by using two-tailed Student’s t-test.

**Figure. S6.**


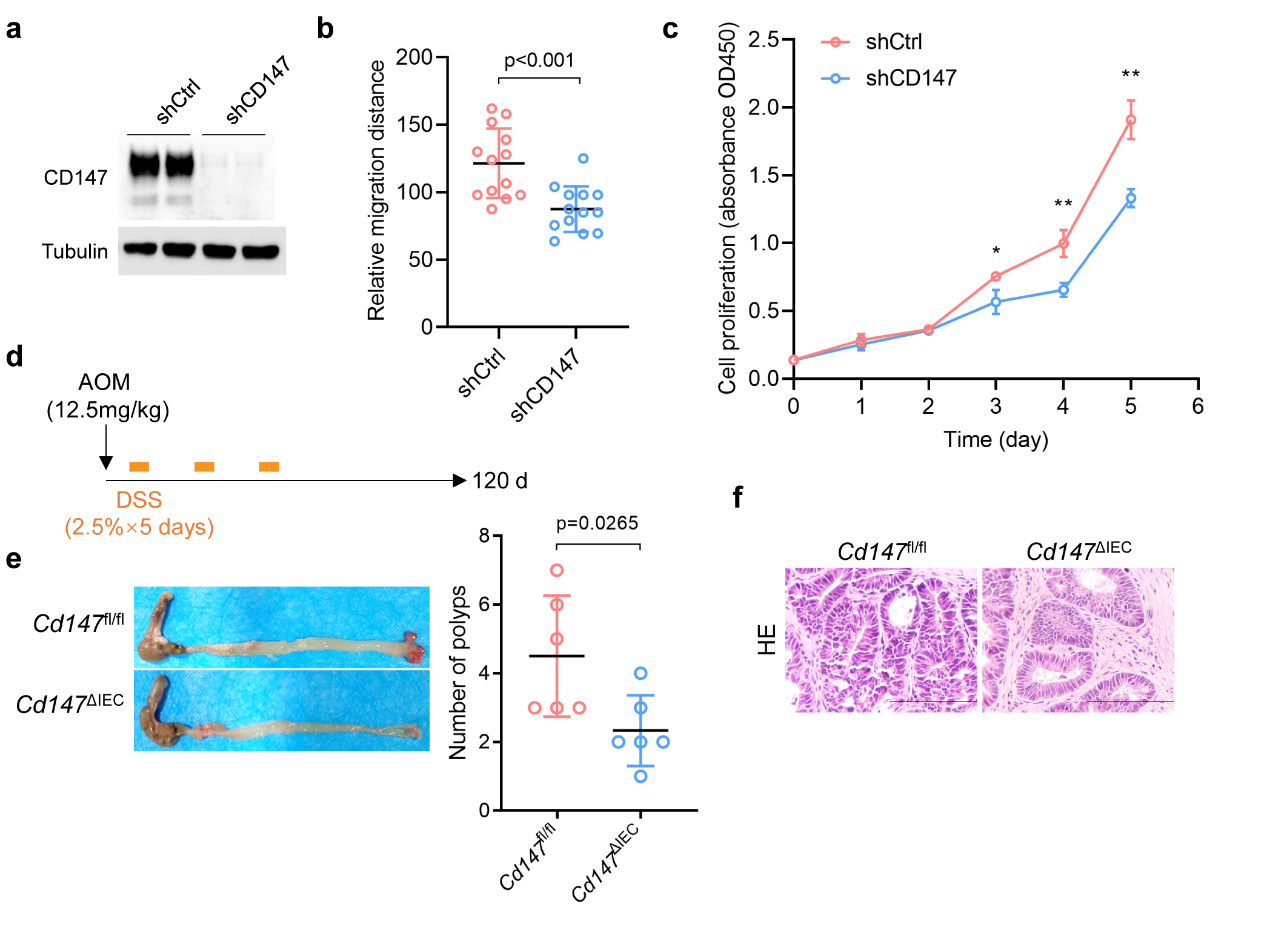


**Supplementary Fig. 6 CD147 deficiency leads to decreased CRC development. a** Western blotting analysis of CD147 expression in HCT116 cells. **b** Quantification of cell migration ability of HCT116 cells. **c** Proliferation curve of HCT116 cells determined by CCK-8 assay. **d** Cohorts of 6 *Cd147^ΔIEC^* mice and 6 *Cd147^fl/fl^* mice were injected i.v. with AOM on day 0 followed by three DSS cycles. **e** Representative macroscopic view of colons from mice of indicated genotypes. The polyps of all the colons were analyzed at the completion of the experiment, and data are presented as a scatter diagram. The p values in (**b**-**c**, **e**) was determined by using Student’s t-test. **f** Photomicrographs of representative sections from the respective groups are shown.

**Figure. S7.**

**
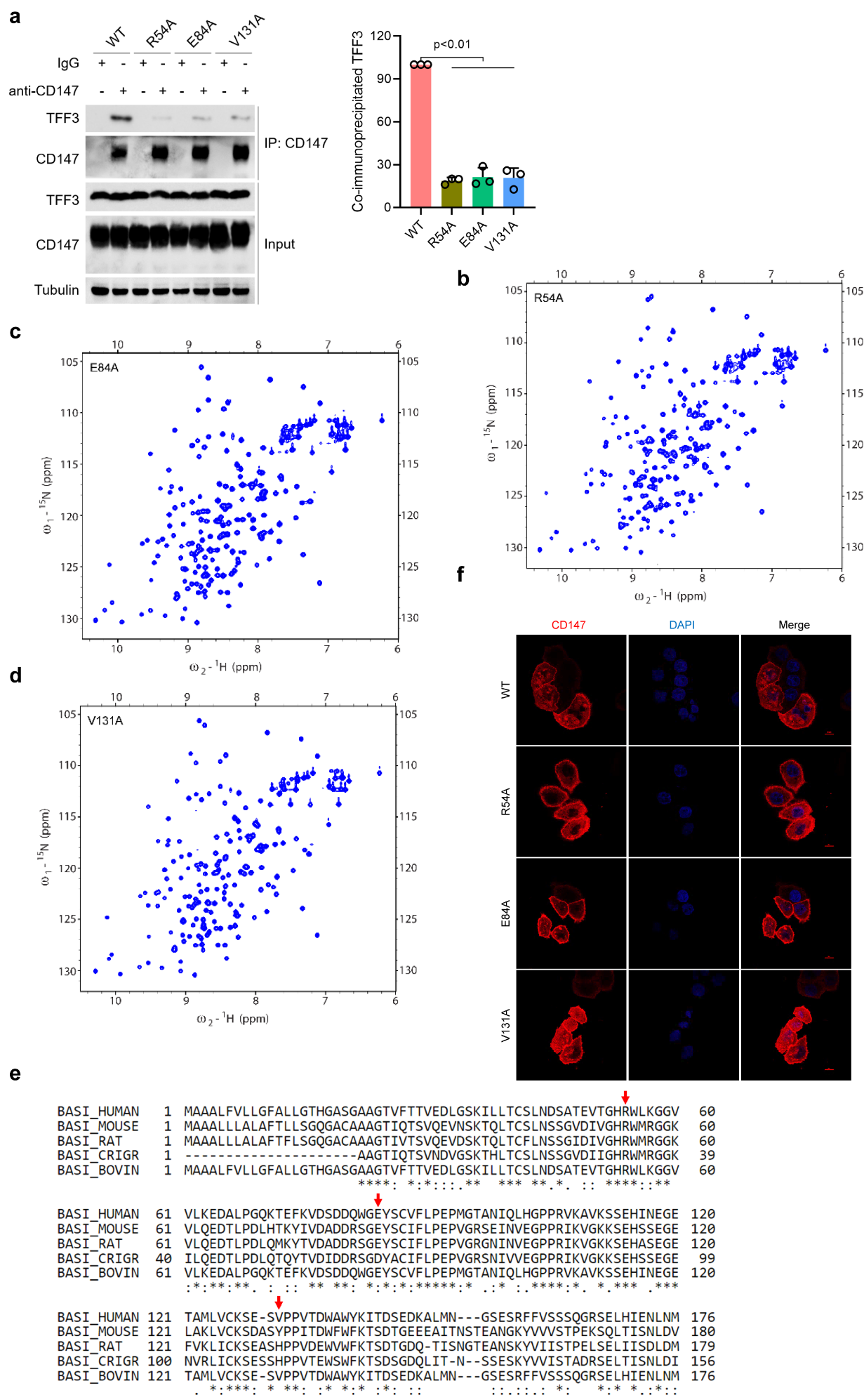
**

**Supplementary Fig. 7 Alanine mutants of the key residues on CD147 for TFF3 binding do not destroy the whole structure of CD147. a** Western blotting analyses of CD147 co-IP with TFF3 in HCT-8 CD147KO cells expressing wild-type (WT), R54A, E84A or V131A CD147. Graph shows semi-quantitative analysis of co-immunoprecipitated TFF3. P value was determined by using two-tailed Student’s t test. **b**-**d** ^15^N-^1^H-HSQC spectra for 0.1 mM CD147^ECD^ mutants. R54A (**b**), E84A (**c**) and V131A (**d**). **e** N-terminal amino acid sequence alignment of human, mouse, rat, Chinese hamster and bovine CD147 residues. Red arrows indicate the key residues on CD147 for TFF3 binding. R54, E84 and V131 in order from top to bottom. **f** Representative confocal images of cellular localization of alanine mutants in HCT-8 CD147KO cells. Scale bar, 10 μm.

**Figure. S8.**

**
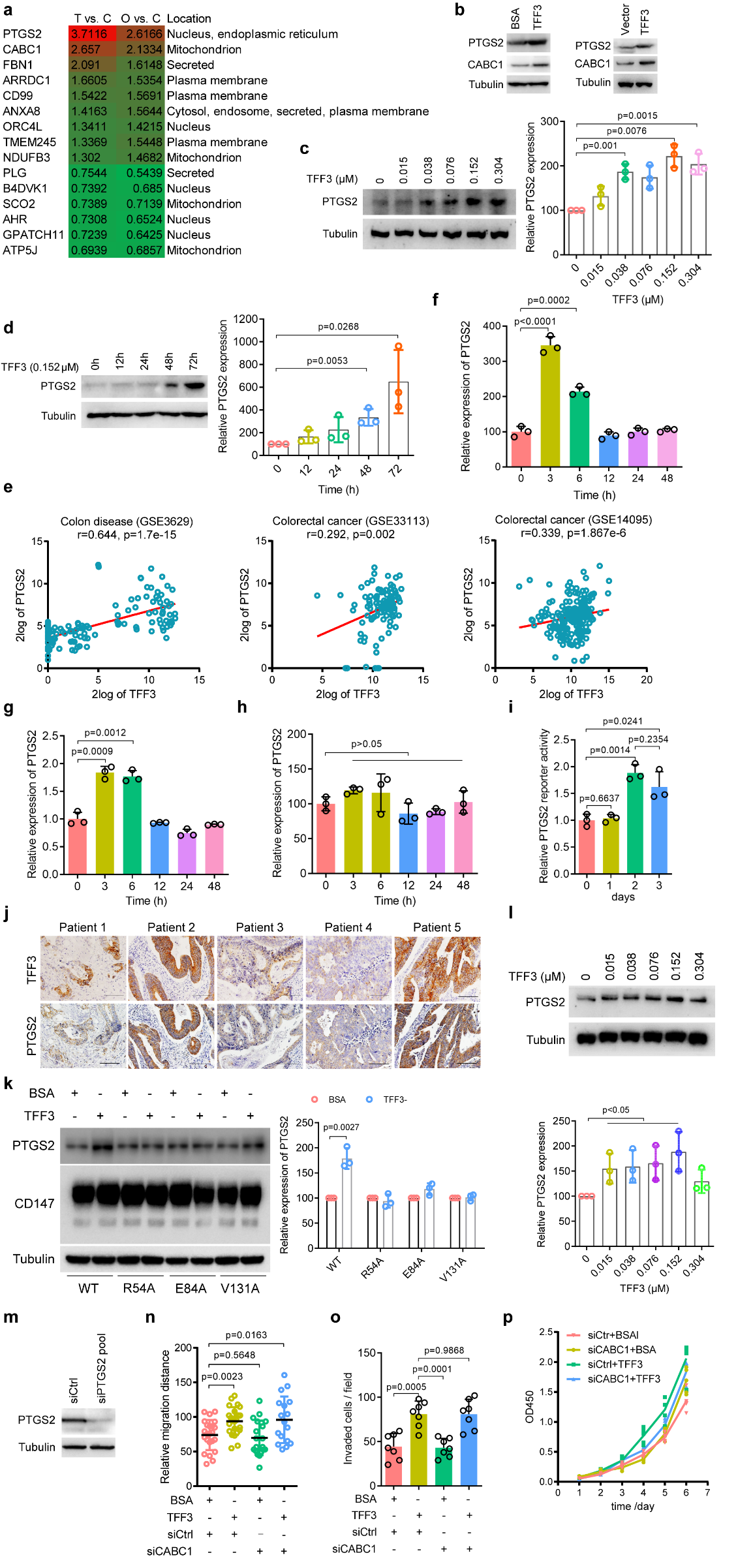
**

**
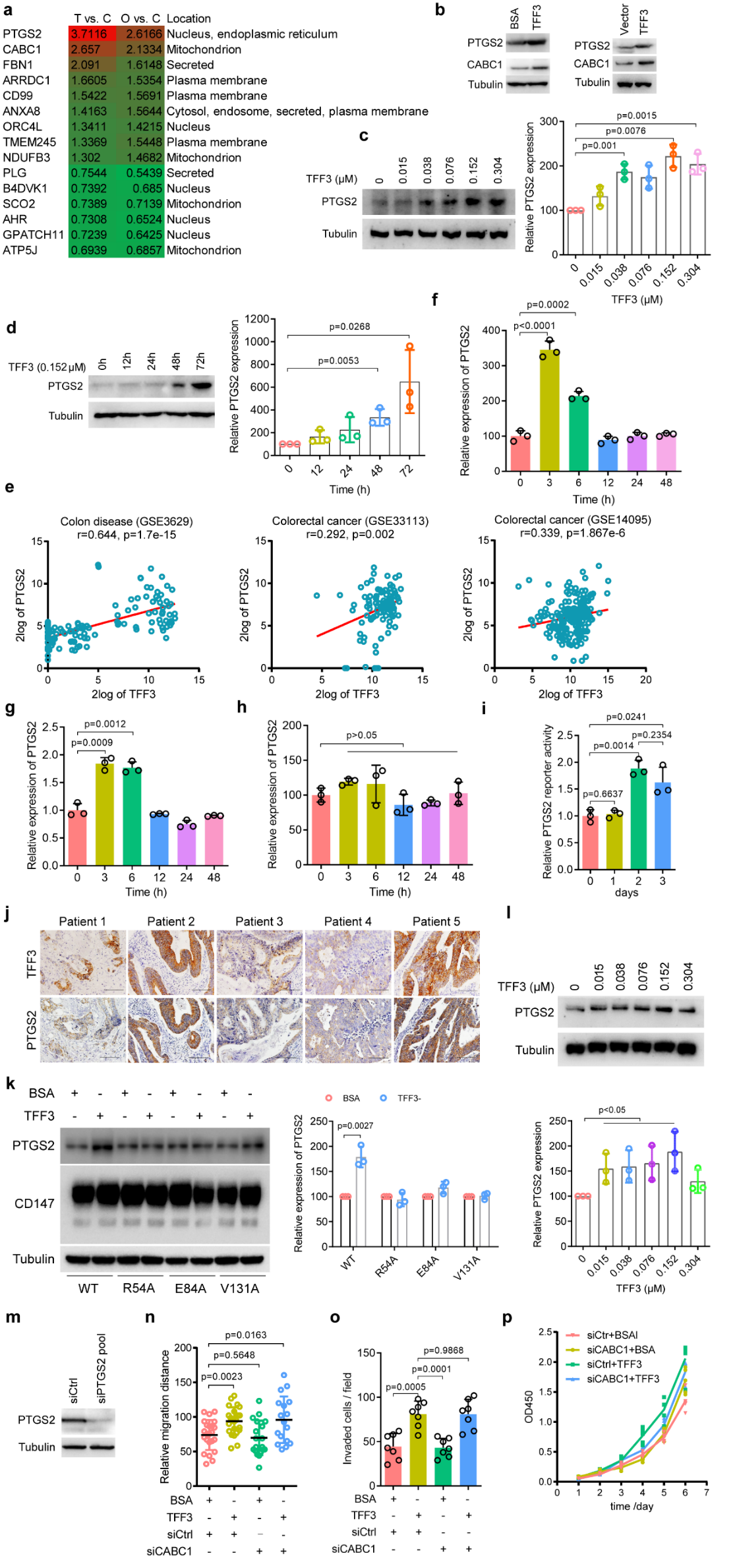
Supplementary Fig. 8 TFF3 promotes migration, invasion and proliferation via PTGS2. a** Heatmap of proteins that were upregulated or downregulated in both groups T (TFF3-treated) and O (TFF3-overexpressing) compared with group C (control). **b** Western blotting analyses of PTGS2 and CABC1 expression in cells treated with TFF3 (left panel) or overexpressing TFF3 (right panel). **c**-**d** Western blotting analyses of PTGS2 expression in HCT-8 cells treated with increasing amounts of TFF3 for 48 h (**c**) or following increasing periods of TFF3 treatment (**d**). Graphs in (**c**-**d**) show semi-quantitative analyses of relative PTGS2 expression. **e** Correlation analyses of gene expression between *TFF3* and *PTGS2* using the GEO data. Pearson correlation coefficients and p-values are shown. **f**-**h** qPCR for *PTGS2* expression normalized to *GAPDH* expression in SW480 (**f**), HCT-8 (**g**) and HCT-8 CD147KO (**h**) cells following increasing periods of 0.152 μM TFF3 treatment. **i** Dual luciferase reporter assays for monitoring *PTGS2* transcription activity in HCT-8 cells following increasing periods of TFF3 treatment. **j** Representative images of immunochemistry staining of TFF3 and PTGS2 in human CRC tissues. Scale bar, 200 μm. **k** Western blotting analyses of the indicated proteins in HCT-8 CD147KO cells. Cells were transfected with WT, R54A, E84A, or V131A CD147 and treated with or without TFF3. Graph shows semi-quantitative analyses of relative PTGS2 expression. **l** Western blotting analysis of PTGS2 expression in FHC cells treated with increasing amounts of TFF3. Graph shows semi-quantitative analyses of relative PTGS2 expression. **m** Western blotting analysis of PTGS2 expression in HCT116 cells transfected with control siRNA or a pool of siRNAs targeting PTGS2. **n**-**p** HCT-8 cells were transfected with siRNA targeting CABC1 (siCABC1) or control siRNA (siCtrl) alone or in combination with TFF3 treatment. (**n**) Cell migration was assessed by wound healing assays. (**o**) Cell invasion was assessed by transwell assays. The graph shows the average number of invaded cells per field. (**p**) Proliferation curves determined by the CCK-8 assay. The p-values in (**c-d**, **f**-**i**, **k**-**l, n-p**) were determined by using two-tailed Student’s t test.

**Figure. S9.**


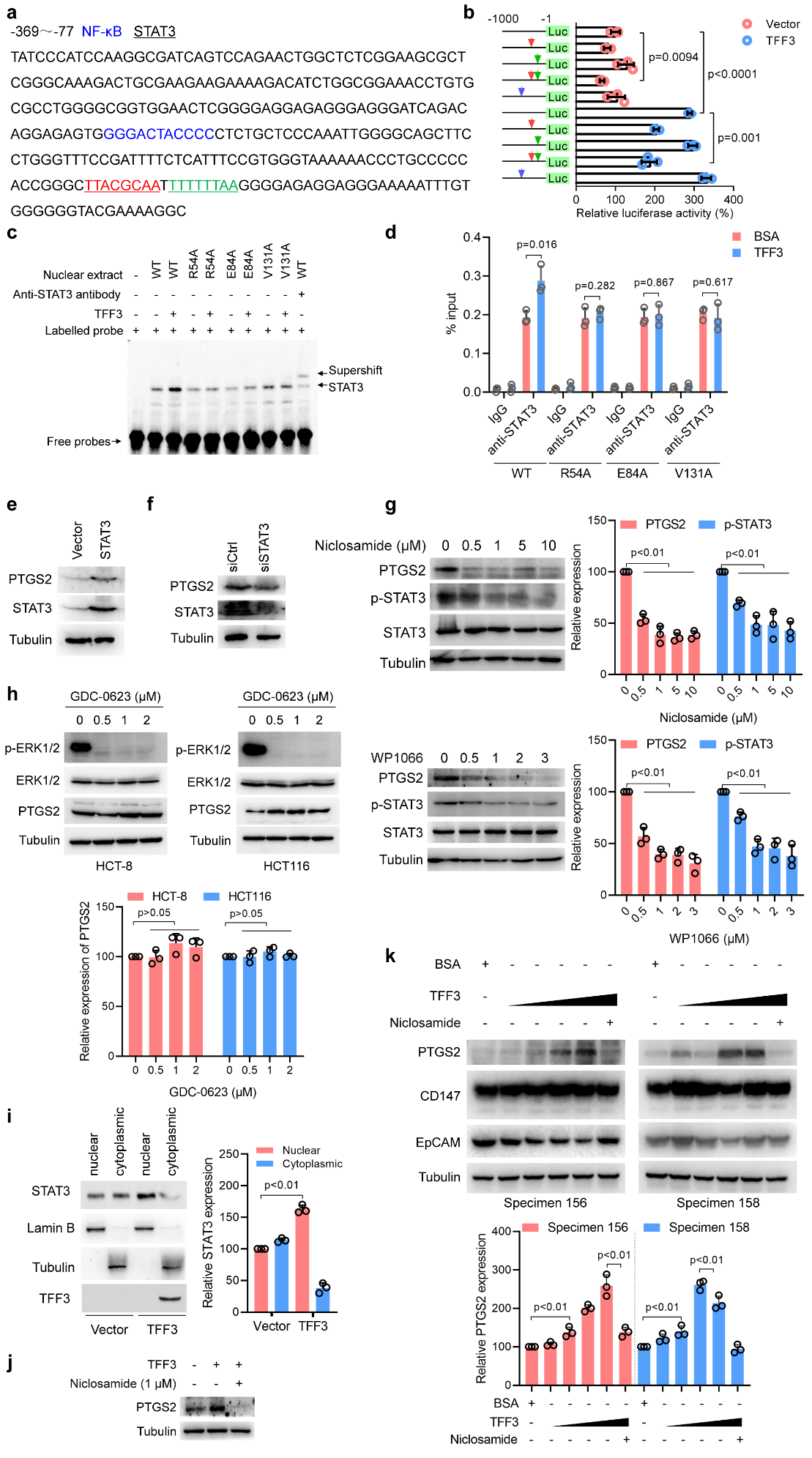


**Supplementary Fig. 9 TFF3 induces PTGS2 expression via promoting the interaction between CD147 and CD44s. a** Sequence of the *PTGS2* promoter spanning −369 to -77 base pairs (bp). Blue letters indicate putative NF-κB binding sites, and red and green letters indicate putative STAT3 binding sites. **b** Schematic representation of the *PTGS2* promoter reporter constructs. HCT-8 cells expressing control plasmid or TFF3 were transfected with the indicated constructs. Blue arrow, mutation of NF-κB binding site; red arrow, mutation of the first STAT3 binding site; green arrow, mutation of the second STAT3 binding site. **c** EMSA to detect the binding of STAT3 to the *PTGS2* promoter in HCT-8 CD147KO cells expressing WT, R54A, E84A, or V131A CD147. **d** ChIP assay for the enrichment of STAT3 at the *PTGS2* promoter in HCT-8 CD147KO cells expressing WT, R54A, E84A, or V131A CD147 in response to TFF3 (0.152 μM， for 24h). The p-values were determined by using two-tailed Student’s t test. **e** Western blotting analyses of the indicated proteins in HCT-8 cells transfected with control vector or STAT3. **f** Western blotting analyses of the indicated proteins in HCT-8 cells transfected with control siRNA (siCtrl) or siRNA targeting STAT3 (siSTAT3). **g** Western blotting analyses of the indicated proteins in HCT-8 cells incubated with increasing amounts of the STAT3 inhibitor niclosamide (upper panel) or WP1066 (lower panel). **h** Western blotting analyses of the indicated proteins in CRC cells incubated with increasing amounts of the MEK1 inhibitor GDC-0623. Graphs in (**g**-**h**) show semi-quantitative analysis of relative PTGS2 expression. **i** Western blotting analyses of indicated proteins in separated nuclear and cytosolic fragments in HCT-8 cells transfected with vector or TFF3. Graph shows semi-quantitative analysis of relative STAT3 expression in separated nuclear and cytosolic fragments. **j** Western blotting analyses of PTGS2 in HCT-8 cells treated with 0.152 μM TFF3 alone or in combination with niclosamide. **k** Western blotting analyses of the indicated proteins in human primary IECs treated with increasing amounts of TFF3 alone or in combination with niclosamide. Graph shows semi-quantitative analysis of relative PTGS2 expression. The p-values in (**b, d**, **g**-**i**, **k**) were determined by using two-tailed Student’s t test.

**Figure. S10.**

**
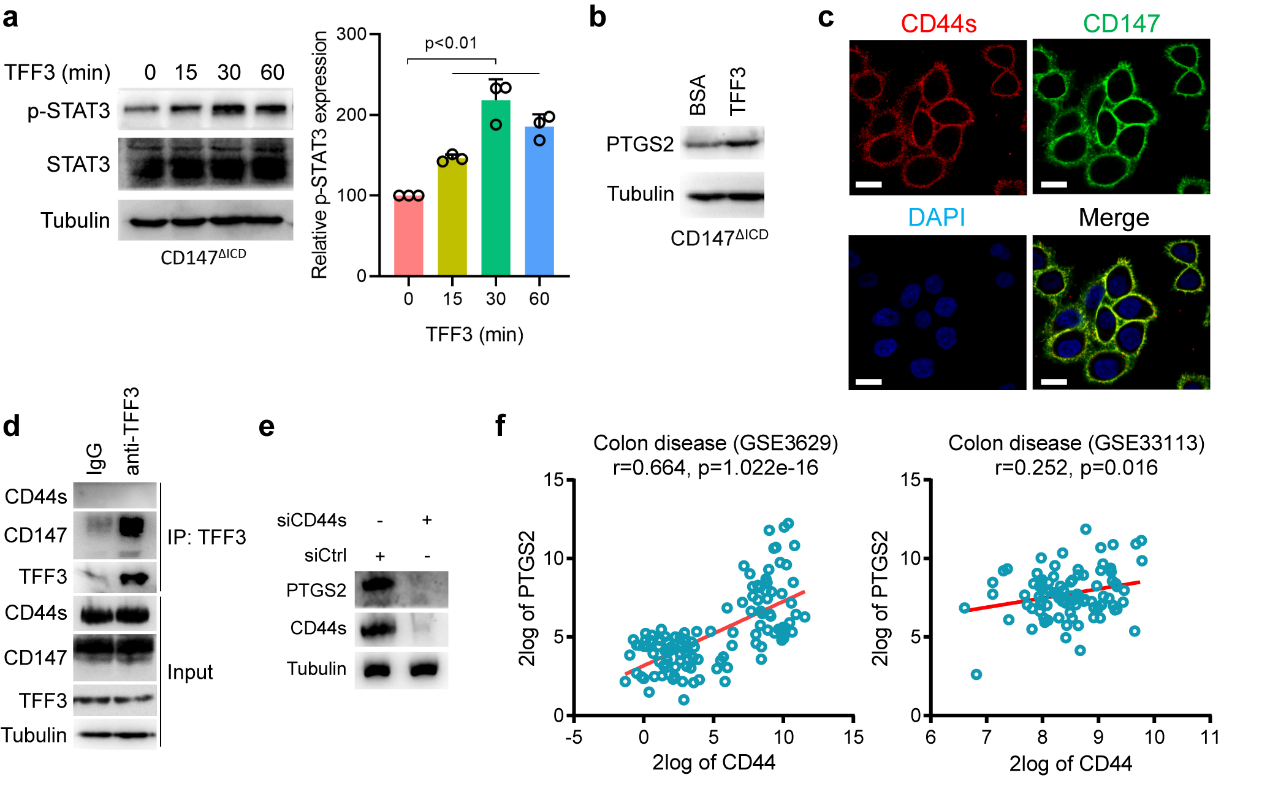
**

**Supplementary Fig. 10 TFF3 promotes the interaction between CD147 and CD44s. a** Western blotting analysis of the indicated proteins in HCT-8 CD147KO cells expressing CD147^ΔICD^ following increasing periods of TFF3 treatment. Graph shows semi-quantitative analysis of relative PTGS2 expression**.** The p-values were determined by using two-tailed Student’s t test. **b** Western blotting analyses of the indicated proteins in HCT-8 CD147KO cells expressing CD147^ΔICD^ treated with BSA or TFF3. **c** Representative images of CD44s and CD147 co-localization in HCT-8 cells. Scale bar, 10 μm. **d** Western blotting analyses of endogenous TFF3 co-IP with CD147 and CD44s in SW620 cells. IgG was used as a control antibody. **e** Western blotting analyses of the indicated proteins in HCT-8 cells transfected with control siRNA (siCtrl) or siRNA targeting CD44s (siCD44s). **f** Correlation analyses of CD44 and PTGS2 gene expression using the GEO data. The p-values were determined by Pearson correlation analysis.

**Figure. S11.**


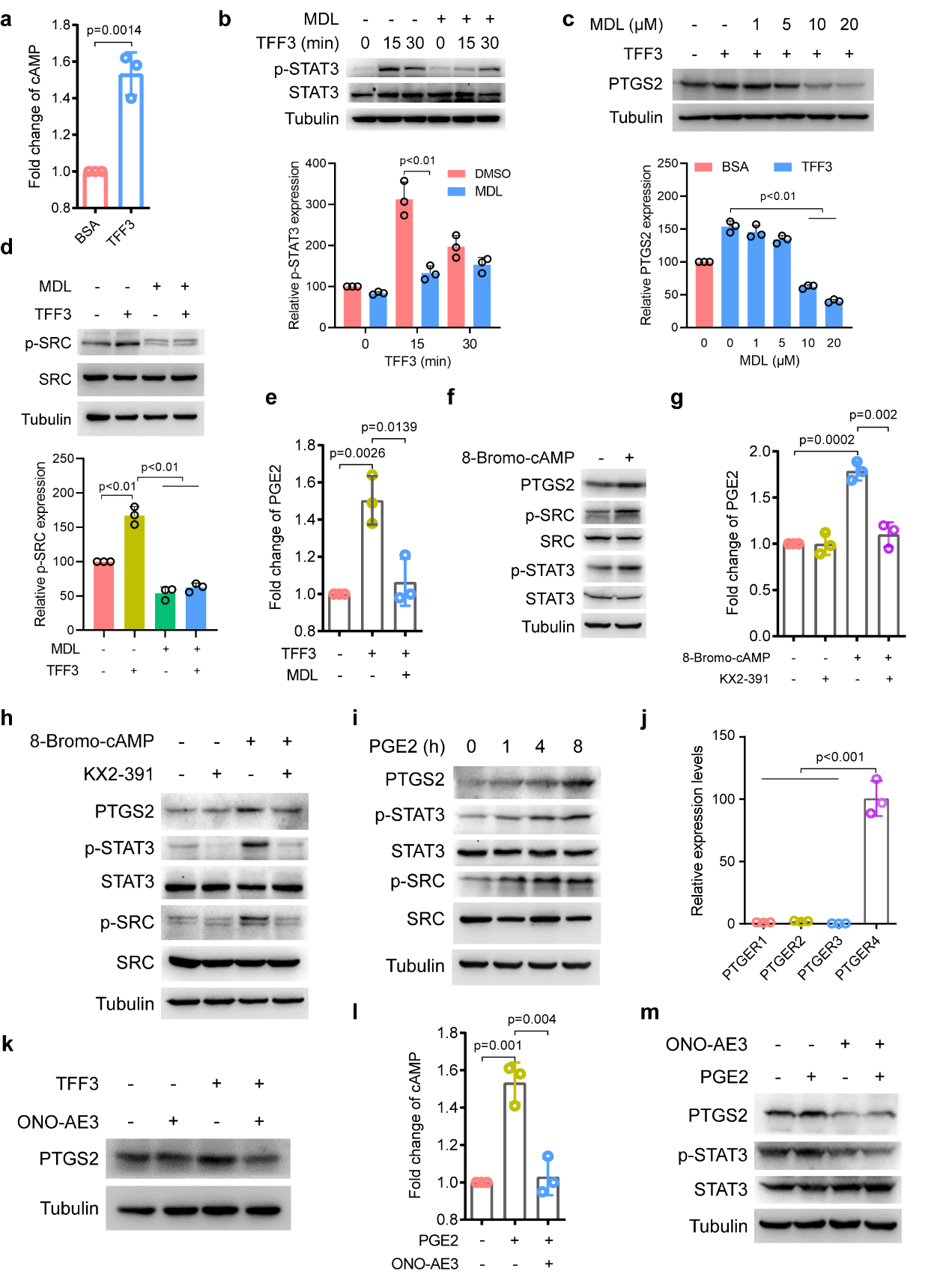


**Supplementary Fig. 11 The PGE2-PTGER4-cAMP-PKA-SRC pathway is a feed-forward loop regulating STAT3 activation and PTGS2 upregulation in CRC cells exposed to TFF3. a** Determination of intracellular cAMP levels in HCT-8 cells following TFF3 treatment. **b** Western blotting analyses of STAT3 activation following increasing periods of TFF3 treatment in the presence or absence of the adenylate cyclase inhibitor MDL-12,330A hydrochloride (MDL). Graph shows semi-quantitative analysis of relative p-STAT3 expression. **c** Western blotting analyses of the indicated proteins in HCT-8 cells treated with TFF3 alone or in combination with increasing amounts of MDL. Graph shows semi-quantitative analysis of relative PTGS2 expression. **d** Western blotting analysis of SRC activation following TFF3 treatment in the presence or absence of MDL. Graph shows semi-quantitative analysis of relative p-SRC expression. **e** Quantification of PGE2 production in HCT-8 cells incubated with TFF3 alone or in combination with MDL. **f** Western blotting analyses of the indicated proteins in HCT-8 cells treated with or without cAMP analogue 8-bromo-cAMP. **g** Quantification of PGE2 production in HCT-8 cells incubated with 8-bromo-cAMP and/or SRC inhibitor KX2-391. **h** Western blotting analyses of the indicated proteins in HCT-8 cells treated with 8-bromo-cAMP and/or KX2-391. **i** Western blotting analyses of the indicated proteins following increasing periods of PGE2 treatment. **j** qPCR analysis of *PGE2 receptor* expression normalized to *GAPDH* expression in HCT-8 cells. **k** Western blotting analysis of PTGS2 expression following TFF3 treatment in the presence or absence of the PTGER4 inhibitor ONO-AE3-208 (ONO-AE3). **l** Determination of intracellular cAMP levels in HCT-8 cells treated with PGE2 alone or in combination with ONO-AE3. **m** Western blotting analyses of the indicated proteins in HCT-8 cells following PGE2 treatment in the presence or absence of ONO-AE3. The p-values in (**b**-**e**, **g**, **j**, **l**) were determined by using two-tailed Student’s t-test.

**Figure. S12.**

**
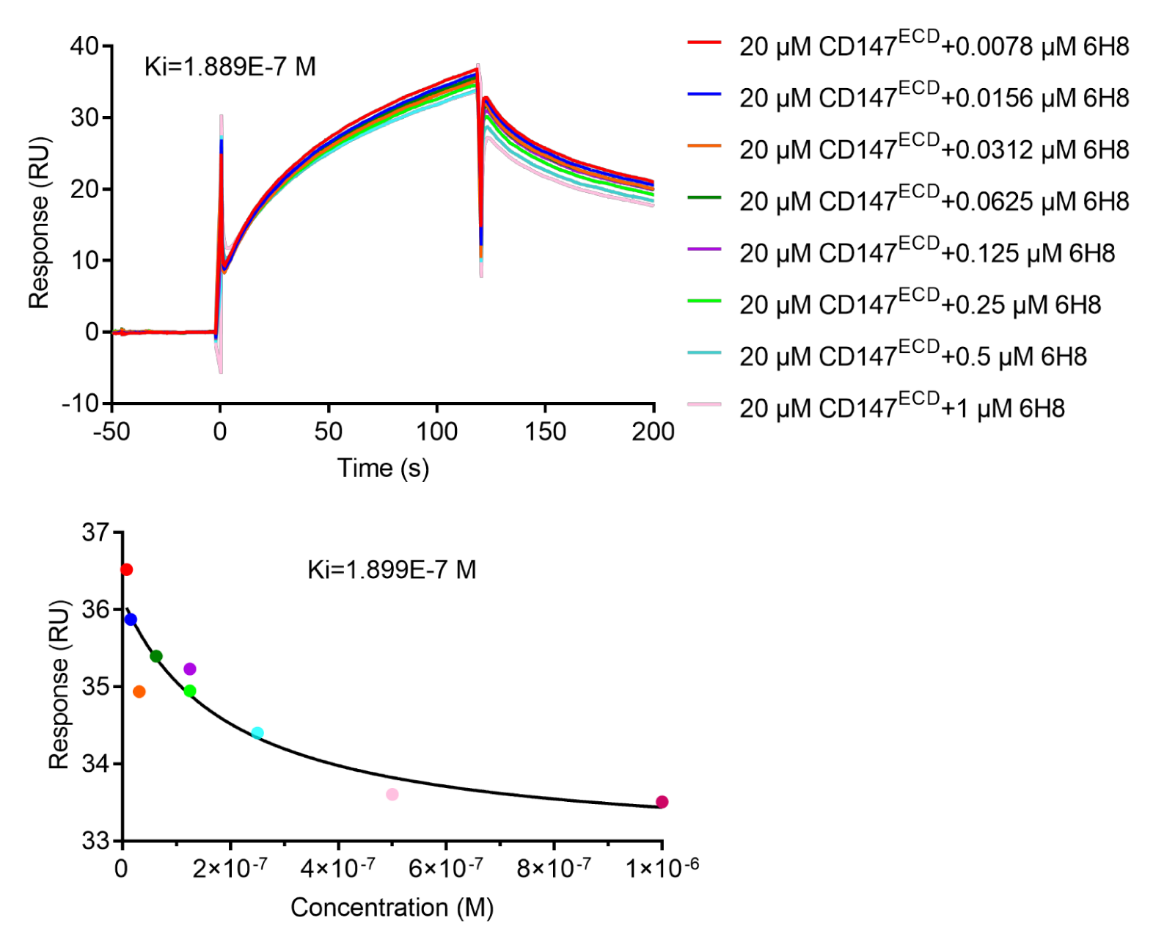
**

**Supplementary Fig. 12 Biophysical analysis of the TFF3-CD147^ECD^ interaction in the presence of the monoclonal antibody 6H8 using SPR.** The indicated concentrations of 6H8 and purified CD147^ECD^ were injected over immobilized TFF3.

**Figure. S13.**


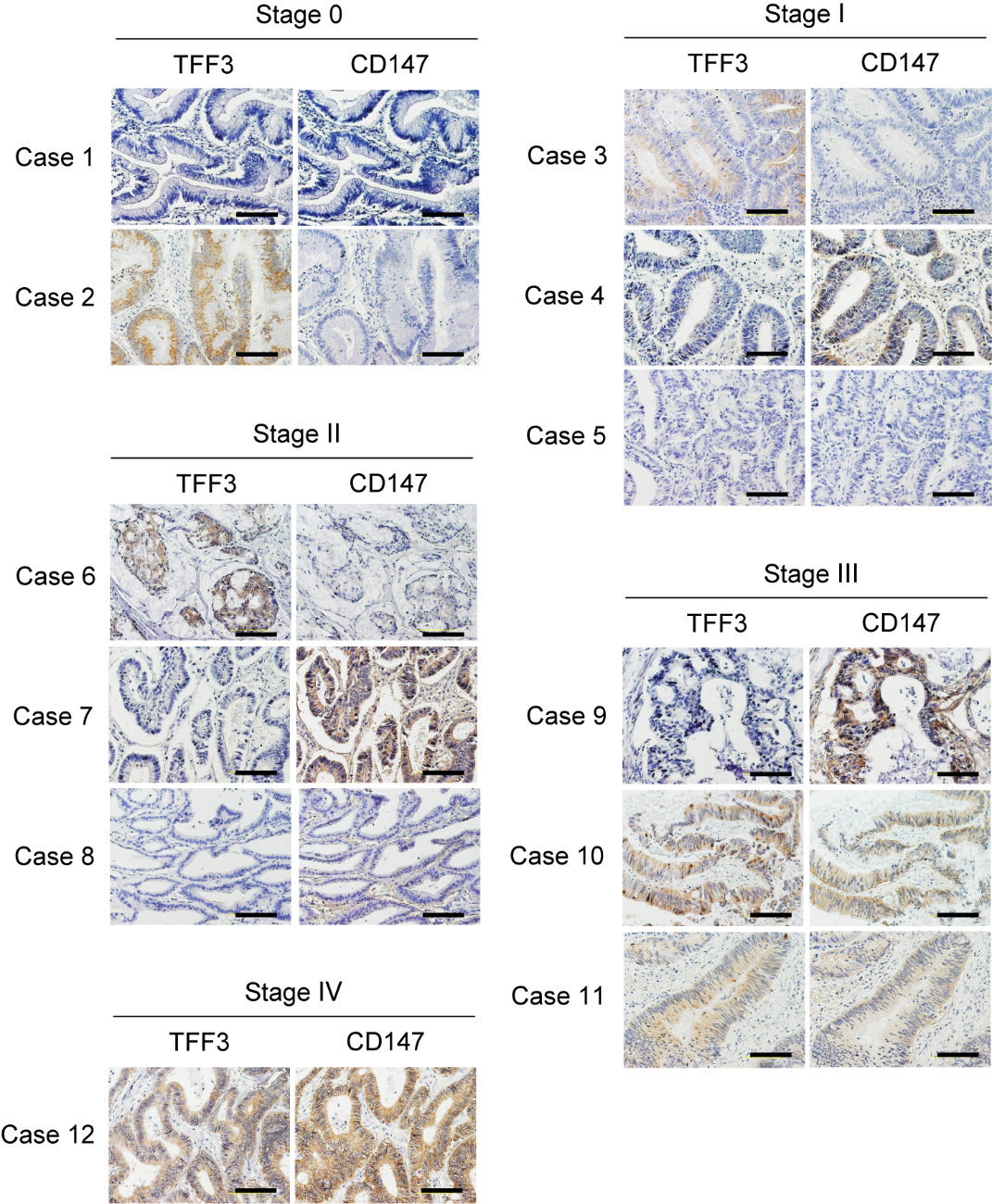
**Supplementary Fig. 13 Immunohistochemical staining of TFF3 and CD147 in CRC specimens.** TFF 3 and CD147 were stained in brown in sequential sections in stage 0 (Case 1 and 2), stage I (Case 3-5), stage II (Case 6-8), stage III (Case 9-11) and stage IV (Case 12). Case 2, 3 and 6, TFF3 positive and CD147 negative; Case 4, 7 and 9, TFF3 negative and CD147 positive; Case 1, 5 and 8, double negative; and Case 10, 11 and 12, double positive. All sections were counterstained with hematoxylin, and representative figures are shown. Scale bars, 200 μm.

| **Supplementary Table 1. Distribution of selected characteristics in colorectal cancer cases and controls.** | | | |
| --- | --- | --- | --- |
| Variables | Cases, n=188 | Controls, n=97 | *p* |
| Sex, No. (%) |  |  |  |
| Male | 101 (53.72) | 53 (54.64) |  |
| Female | 87 (46.28) | 44 (45.36) | 0.883^a)^ |
| Smoking status, No. (%) |  |  |  |
| Never | 114 (60.64) | 63 (64.95) |  |
| Ever | 74 (39.36) | 34 (35.05) | 0.477 ^a)^ |
| Alcohol consumption, No. (%) |  |  |  |
| Never | 161 (85.64) | 85 (87.63) |  |
| Ever | 27 (14.36) | 12 (12.37) | 0.643 ^a)^ |
| Education level |  |  |  |
| Up to middle school diploma | 119 (63.30) | 54 (55.67) |  |
| High school or equivalent | 46 (24.47) | 24 (24.74) |  |
| College degree or higher | 23 (12.23) | 19 (19.59) | 0.227 ^a)^ |
| History of NSAID use, No. (%) |  |  |  |
| Yes | 3 (1.60) | 1 (1.03) |  |
| No | 185 (98.40) | 96 (98.97) | 1.000 ^b)^ |
| Age, [year] mean (SD) | 56.12 (11.21) | 57.02 (12.03) | 0.532 ^c)^ |
| Serum TFF3 [pg/ml]  median (range) | 3226  (1502-11939) | 2083  (964.6-7255) | <0.0001 ^c)^ |

SD, standard deviation.

^a)^ p values by Pearson's Chi-square test.

^b)^ p value by Fisher's exact test.

^c)^ p values by Student’s t test.

| **Supplementary Table 2. Serum TFF3 and TFF3 expression in corresponding CRC tissues.** | | |
| --- | --- | --- |
| Patient ID | IHC score | Serum TFF3 (pg/mL) |
| 453 | 0 | 1993.33 |
| 462 | 0 | 1501.67 |
| 470 | 0 | 2789.17 |
| 481 | 1 | 3330.83 |
| 490 | 1 | 1130.83 |
| 491 | 1 | 3376.67 |
| 497 | 0 | 3793.33 |
| 499 | 2 | 4205.83 |
| 503 | 3 | 5491.25 |
| 506 | 0 | 2710.00 |
| 521 | 3 | 5278.75 |
| 526 | 2 | 4766.25 |
| 528 | 0 | 1641.25 |
| 535 | 1 | 5610.00 |
| 541 | 0 | 3441.25 |
| 547 | 0 | 4360.00 |
| 548 | 0 | 2041.25 |
| 550 | 0 | 2185.00 |
| 552 | 1 | 2753.75 |
| 565 | 1 | 6139.17 |
| 567 | 2 | 3318.33 |
| 571 | 1 | 2497.50 |
| 579 | 1 | 2439.17 |
| 584 | 1 | 4160.00 |
| 590 | 0 | 2043.33 |
| 631 | 1 | 2987.78 |
| 634 | 2 | 3910.00 |
| 638 | 2 | 2304.44 |
| 647 | 1 | 3321.11 |
| 672 | 1 | 2310.00 |
| 673 | 0 | 1293.33 |
| 676 | 0 | 3193.33 |
| 680 | 2 | 4993.33 |
| 683 | 3 | 2998.89 |
| 692 | 3 | 3382.22 |
| 695 | 0 | 2693.33 |
| 696 | 0 | 1232.22 |
| 697 | 1 | 2898.89 |
| 699 | 3 | 6711.11 |
| 711 | 1 | 2175.33 |
| 712 | 1 | 2378.67 |
| 731 | 1 | 5142.00 |
| 766 | 1 | 3408.67 |
| 787 | 1 | 3868.67 |

ρ=0.538, p=1.65E-04, n=44, non-parametric Spearman correlation

| **Supplementary Table 3. Protein sequence confirmation of purified TFF3 by MS** | | | | | | | | | | | | |
| --- | --- | --- | --- | --- | --- | --- | --- | --- | --- | --- | --- | --- |
| Description | Coverage | # PSMs | | # Peptides | | # AAs | | |  |  |  |  |
| TFF3 | 69.49 | 167 | | 8 | | 59 | | |  |  |  |  |
| Sequence | | | Probability | | Area | | m/z [Da] | MH+ [Da] | | ΔM [ppm] | RT [min] | Ions Matched |
| DRVDcGYPHVTPKEcNNR | | | 98.54 | | 2.747E8 | | 555.00287 | 2216.98964 | | -1.28 | 19.48 | 36/102 |
| DRVDcGYPHVTPKEcNNR | | | 105.08 | | 2.747E8 | | 555.00275 | 2216.98916 | | -1.50` | 20.62 | 32/102 |
| DRVDcGYPHVTPKEcNNR | | | 101.17 | | 2.747E8 | | 555.00330 | 2216.99135 | | -0.51 | 18.64 | 38/102 |
| DRVDcGYPHVTPKEcNNR | | | 92.67 | | 2.747E8 | | 555.00299 | 2216.99013 | | -1.06 | 19.20 | 37/102 |
| DRVDcGYPHVTPKEcNNR | | | 90.12 | | 2.747E8 | | 555.00281 | 2216.98940 | | -1.39 | 20.20 | 32/102 |
| DRVDcGYPHVTPKEcNNR | | | 82.85 | | 2.747E8 | | 555.00269 | 2216.98891 | | -1.61 | 19.36 | 34/102 |
| DRVDcGYPHVTPKEcNNR | | | 81.88 | | 2.747E8 | | 555.00311 | 2216.99062 | | -0.84 | 19.80 | 37/102 |
| DRVDcGYPHVTPKEcNNR | | | 104.36 | | 2.747E8 | | 555.00397 | 2216.99404 | | 0.70 | 18.92 | 33/102 |
| DRVDcGYPHVTPKEcNNR | | | 66.12 | | 2.725E7 | | 739.66785 | 2216.98899 | | -1.58 | 18.73 | 26/68 |
| DRVDcGYPHVTPK | | | 63.36 | | 1.113E9 | | 515.24768 | 1543.72849 | | -2.43 | 19.06 | 27/48 |
| DRVDcGYPHVTPKEcNNR | | | 101.72 | | 2.747E8 | | 555.00330 | 2216.99135 | | -0.51 | 20.89 | 33/102 |
| DRVDcGYPHVTPKEcNNR | | | 124.13 | | 2.747E8 | | 555.00287 | 2216.98964 | | -1.28 | 20.53 | 34/102 |
| DRVDcGYPHVTPK | | | 77.57 | | 1.113E9 | | 515.24762 | 1543.72831 | | -2.55 | 19.47 | 27/48 |
| VDcGYPHVTPKEcNnRGccFDSR | | | 45.06 | |  | | 708.04681 | 2829.16543 | | 2.02 | 23.83 | 30/132 |
| DRVDcGYPHVTPKEcNNR | | | 50.98 | | 2.725E7 | | 739.66943 | 2216.99375 | | 0.57 | 19.61 | 25/68 |
| DRVDcGYPHVTPKEcNNR | | | 71.67 | | 2.725E7 | | 739.66797 | 2216.98935 | | -1.41 | 18.59 | 24/68 |
| VDcGYPHVTPKEcNnRGccFDSR | | | 59.73 | |  | | 708.04736 | 2829.16762 | | 2.79 | 22.28 | 31/132 |
| DRVDcGYPHVTPKEcNNR | | | 67.89 | |  | | 555.00336 | 2216.99160 | | -0.40 | 21.79 | 26/102 |
| DRVDcGYPHVTPKEcNNR | | | 39.34 | | 2.725E7 | | 739.66797 | 2216.98935 | | -1.41 | 17.88 | 24/68 |
| VDcGYPHVTPKEcnNRGccFDSR | | | 45.06 | |  | | 708.04681 | 2829.16543 | | 2.02 | 23.83 | 29/132 |
| VDcGYPHVTPK | | | 49.15 | | 1.840E9 | | 636.80524 | 1272.60320 | | -0.77 | 19.79 | 16/20 |
| VDcGYPHVTPKEcNNR | | | 99.22 | | 5.204E8 | | 649.29211 | 1945.86179 | | -1.35 | 19.15 | 27/60 |
| VDcGYPHVTPKEcNNR | | | 102.55 | | 5.204E8 | | 649.29199 | 1945.86142 | | -1.53 | 18.79 | 29/60 |
| VDcGYPHVTPK | | | 42.23 | | 3.516E9 | | 424.87344 | 1272.60578 | | 1.26 | 20.93 | 21/40 |
| EcNNRGccFDSR | | | 57.83 | | 8.152E8 | | 525.53387 | 1574.58707 | | -1.46 | 17.86 | 18/44 |
| VDcGYPHVTPK | | | 41.43 | |  | | 424.87241 | 1272.60266 | | -1.19 | 25.06 | 19/40 |
| VDcGYPHVTPK | | | 34.32 | | 3.516E9 | | 424.87326 | 1272.60523 | | 0.83 | 18.83 | 20/40 |
| VDcGYPHVTPKEcNNR | | | 32.67 | |  | | 487.22083 | 1945.86147 | | -1.51 | 21.16 | 32/90 |
| VDcGYPHVTPKEcNNR | | | 62.53 | | 5.784E8 | | 487.22147 | 1945.86403 | | -0.19 | 18.84 | 36/90 |
| VDcGYPHVTPKEcnNRGccFDSR | | | 59.73 | |  | | 708.04736 | 2829.16762 | | 2.79 | 22.28 | 31/132 |
| EcNNRGccFDSR | | | 60.12 | |  | | 525.53424 | 1574.58817 | | -0.76 | 18.27 | 17/44 |
| VDcGYPHVTPK | | | 47.12 | |  | | 424.87289 | 1272.60413 | | -0.04 | 21.96 | 20/40 |
| VDcGYPHVTPK | | | 81.49 | | 1.840E9 | | 636.80493 | 1272.60259 | | -1.25 | 18.77 | 17/20 |
| VDcGYPHVTPK | | | 68.26 | |  | | 636.80475 | 1272.60222 | | -1.54 | 22.89 | 15/20 |
| EcNNRGccFDSR | | | 56.21 | |  | | 525.53314 | 1574.58487 | | -2.86 | 19.22 | 20/44 |
| VDcGYPHVTPKEcNNR | | | 81.18 | | 5.784E8 | | 487.22101 | 1945.86220 | | -1.13 | 18.67 | 29/90 |
| VDcGYPHVTPK | | | 46.17 | | 9.774E6 | | 636.80566 | 1272.60405 | | -0.10 | 26.03 | 14/20 |
| DRVDcGYPHVTPKEcNNR | | | 52.85 | |  | | 555.00562 | 2217.00063 | | 3.68 | 22.34 | 27/102 |
| EcNNRGccFDSR | | | 51.35 | | 8.152E8 | | 525.53357 | 1574.58615 | | -2.04 | 16.38 | 19/44 |
| VDcGYPHVTPKEcNNR | | | 54.77 | |  | | 487.22171 | 1945.86501 | | 0.31 | 20.87 | 30/90 |
| VDcGYPHVTPKEcNnRGccFDSR | | | 54.01 | |  | | 708.04742 | 2829.16787 | | 2.88 | 21.64 | 32/132 |
| VDcGYPHVTPKEcNNR | | | 89.59 | | 5.784E8 | | 487.22040 | 1945.85976 | | -2.39 | 19.84 | 31/90 |
| EcNNRGccFDSR | | | 68.20 | | 8.152E8 | | 525.53394 | 1574.58725 | | -1.35 | 17.25 | 17/44 |
| EEYVGLSANQcAVPAKDRVDcG  YPHVTPK | | | 41.40 | | 1.165E8 | | 652.91553 | 3260.54853 | | 0.64 | 25.13 | 35/224 |
| VDcGYPHVTPK | | | 37.42 | |  | | 424.87286 | 1272.60404 | | -0.11 | 22.99 | 21/40 |
| VDcGYPHVTPKEcNNR | | | 54.83 | | 5.784E8 | | 487.22025 | 1945.85915 | | -2.70 | 18.57 | 35/90 |
| VDcGYPHVTPK | | | 43.91 | | 3.516E9 | | 424.87292 | 1272.60422 | | 0.04 | 19.90 | 21/40 |
| VDcGYPHVTPKEcNNR | | | 69.42 | | 5.784E8 | | 487.22137 | 1945.86367 | | -0.38 | 19.03 | 34/90 |
| EcNNRGccFDSR | | | 58.39 | | 8.152E8 | | 525.53400 | 1574.58744 | | -1.23 | 15.88 | 18/44 |
| EcNNRGccFDSR | | | 46.67 | |  | | 525.53357 | 1574.58615 | | -2.04 | 20.50 | 16/44 |
| VDcGYPHVTPK | | | 58.22 | | 1.840E9 | | 636.80646 | 1272.60564 | | 1.15 | 20.84 | 15/20 |
| VDcGYPHVTPKEcNNR | | | 78.91 | | 5.784E8 | | 487.22214 | 1945.86672 | | 1.19 | 20.32 | 33/90 |
| VDcGYPHVTPKEcNNR | | | 35.78 | |  | | 487.22055 | 1945.86037 | | -2.07 | 22.16 | 24/90 |
| VDcGYPHVTPK | | | 26.73 | | 3.516E9 | | 424.87292 | 1272.60422 | | 0.04 | 17.79 | 17/40 |
| DRVDcGYPHVTPK | | | 42.70 | | 6.530E7 | | 772.37134 | 1543.73540 | | 2.04 | 20.09 | 15/24 |
| VDcGYPHVTPKEcNNR | | | 77.26 | | 5.784E8 | | 487.22101 | 1945.86220 | | -1.13 | 19.32 | 29/90 |
| VDcGYPHVTPKEcNNR | | | 111.88 | | 5.204E8 | | 649.29297 | 1945.86435 | | -0.03 | 18.25 | 27/60 |
| EcNNRGccFDSR | | | 56.74 | |  | | 525.53436 | 1574.58854 | | -0.53 | 20.22 | 15/44 |
| EcNNRGccFDSR | | | 45.70 | |  | | 525.53314 | 1574.58487 | | -2.86 | 18.58 | 17/44 |
| VDcGYPHVTPKEcnNRGccFDSR | | | 54.01 | |  | | 708.04742 | 2829.16787 | | 2.88 | 21.64 | 33/132 |
| EcNNRGccFDSR | | | 58.95 | |  | | 525.53375 | 1574.58670 | | -1.70 | 20.99 | 18/44 |
| VDcGYPHVTPKEcNNR | | | 113.36 | | 5.204E8 | | 649.29199 | 1945.86142 | | -1.53 | 19.31 | 27/60 |
| VDcGYPHVTPKEcNNR | | | 56.66 | |  | | 487.22095 | 1945.86196 | | -1.26 | 21.31 | 31/90 |
| DRVDcGYPHVTPK | | | 48.51 | |  | | 515.24792 | 1543.72922 | | -1.96 | 18.03 | 21/48 |
| VDcGYPHVTPKEcNNR | | | 101.38 | | 5.784E8 | | 487.22058 | 1945.86049 | | -2.01 | 19.64 | 32/90 |
| VDcGYPHVTPK | | | 82.78 | |  | | 636.80536 | 1272.60344 | | -0.58 | 21.87 | 16/20 |
| VDcGYPHVTPK | | | 57.70 | |  | | 636.80432 | 1272.60137 | | -2.21 | 24.90 | 14/20 |
| VDcGYPHVTPKEcNNR | | | 87.39 | | 5.784E8 | | 487.21967 | 1945.85683 | | -3.89 | 18.43 | 37/90 |
| VDcGYPHVTPKEcNNR | | | 36.93 | |  | | 487.22168 | 1945.86489 | | 0.25 | 21.66 | 33/90 |
| DRVDcGYPHVTPKEcNNR | | | 51.81 | | 2.747E8 | | 555.00342 | 2216.99184 | | -0.29 | 17.60 | 31/102 |
| VDcGYPHVTPKEcNNR | | | 53.10 | | 5.784E8 | | 487.22131 | 1945.86342 | | -0.51 | 20.66 | 35/90 |
| VDcGYPHVTPKEcNNR | | | 57.60 | | 5.784E8 | | 487.22110 | 1945.86257 | | -0.95 | 19.18 | 32/90 |
| DRVDcGYPHVTPK | | | 21.26 | | 1.422E8 | | 386.68781 | 1543.72939 | | -1.85 | 18.44 | 28/72 |
| EcNNRGccFDSR | | | 56.74 | |  | | 525.53357 | 1574.58615 | | -2.04 | 18.68 | 16/44 |
| EcNNRGccFDSR | | | 50.94 | |  | | 525.53381 | 1574.58689 | | -1.58 | 21.42 | 18/44 |
| VDcGYPHVTPKEcNNR | | | 41.42 | |  | | 487.22083 | 1945.86147 | | -1.51 | 22.60 | 22/90 |
| VDcGYPHVTPK | | | 27.26 | |  | | 424.87225 | 1272.60221 | | -1.55 | 26.10 | 17/40 |
| EcNNRGccFDSR | | | 67.45 | | 8.152E8 | | 525.53394 | 1574.58725 | | -1.35 | 16.74 | 19/44 |
| EcNNRGccFDSR | | | 43.87 | |  | | 525.53363 | 1574.58634 | | -1.93 | 20.38 | 15/44 |
| VDcGYPHVTPKEcNNR | | | 33.28 | |  | | 487.22046 | 1945.86001 | | -2.26 | 22.92 | 30/90 |
| VDcGYPHVTPKEcNnRGccFDSR | | | 27.87 | | 5.318E7 | | 566.63922 | 2829.16700 | | 2.57 | 21.56 | 43/176 |
| EcNNRGccFDSR | | | 57.28 | | 8.152E8 | | 525.53375 | 1574.58670 | | -1.70 | 17.62 | 15/44 |
| VDcGYPHVTPKEcNnRGccFDSR | | | 27.03 | |  | | 708.04840 | 2829.17177 | | 4.26 | 22.59 | 24/132 |
| VDcGYPHVTPKEcNNR | | | 24.06 | | 5.784E8 | | 487.21988 | 1945.85769 | | -3.45 | 17.31 | 26/90 |
| EcNNRGccFDSR | | | 64.59 | |  | | 525.53290 | 1574.58414 | | -3.32 | 23.35 | 15/44 |
| DRVDcGYPHVTPK | | | 26.54 | | 1.422E8 | | 386.68903 | 1543.73427 | | 1.31 | 19.44 | 29/72 |
| EcNNRGccFDSR | | | 57.83 | |  | | 525.53296 | 1574.58432 | | -3.21 | 22.48 | 15/44 |
| EcNNRGccFDSR | | | 59.53 | |  | | 787.79895 | 1574.59062 | | 0.79 | 18.52 | 11/22 |
| VDcGYPHVTPK | | | 42.64 | |  | | 424.87225 | 1272.60221 | | -1.55 | 23.99 | 17/40 |
| VDcGYPHVTPKEcNNR | | | 23.14 | |  | | 487.22131 | 1945.86342 | | -0.51 | 23.53 | 28/90 |
| EEYVGLSANQcAVPAKDRVDcG  YPHVTPK | | | 20.03 | | 1.165E8 | | 652.91425 | 3260.54212 | | -1.32 | 25.39 | 38/224 |
| VDcGYPHVTPKEcNnRGccFDSR | | | 10.06 | | 5.318E7 | | 566.64166 | 2829.17921 | | 6.89 | 21.44 | 44/176 |
| EcNNRGccFDSR | | | 24.22 | |  | | 525.53326 | 1574.58524 | | -2.63 | 22.09 | 15/44 |
| EcNNRGccFDSR | | | 18.87 | | 1.420E8 | | 787.79810 | 1574.58891 | | -0.29 | 17.91 | 12/22 |
| VDcGYPHVTPK | | | 40.38 | | 9.774E6 | | 636.80609 | 1272.60491 | | 0.57 | 25.73 | 12/20 |
| VDcGYPHVTPKEcNNR | | | 69.44 | | 1.532E7 | | 973.43701 | 1945.86675 | | 1.20 | 17.70 | 15/30 |
| EcNNRGccFDSR | | | 38.84 | |  | | 525.53314 | 1574.58487 | | -2.86 | 21.28 | 16/44 |
| VDcGYPHVTPKEcnNRGccFDSR | | | 10.06 | | 5.318E7 | | 566.64166 | 2829.17921 | | 6.89 | 21.44 | 43/176 |
| VDcGYPHVTPKEcnNRGccFDSR | | | 20.42 | | 5.318E7 | | 566.63922 | 2829.16700 | | 2.57 | 21.56 | 43/176 |
| DRVDcGYPHVTPKEcNNR | | | 48.90 | |  | | 555.00433 | 2216.99550 | | 1.36 | 22.86 | 27/102 |
| VDcGYPHVTPK | | | 52.50 | | 9.774E6 | | 636.80396 | 1272.60063 | | -2.78 | 26.23 | 13/20 |
| GccFDSR | | | 50.33 | | 2.239E9 | | 451.16766 | 901.32805 | | -1.21 | 14.94 | 10/12 |
| EcNNRGccFDSR | | | 28.62 | |  | | 525.53339 | 1574.58561 | | -2.39 | 23.67 | 17/44 |
| VDcGYPHVTPK | | | 35.90 | | 1.840E9 | | 636.80615 | 1272.60503 | | 0.67 | 17.76 | 12/20 |
| DRVDcGYPHVTPK | | | 49.60 | | 6.530E7 | | 772.37152 | 1543.73577 | | 2.28 | 19.45 | 14/24 |
| VDcGYPHVTPKEcnNRGccFDSR | | | 27.03 | |  | | 708.04840 | 2829.17177 | | 4.26 | 22.59 | 24/132 |
| VDcGYPHVTPK | | | 52.57 | | 9.774E6 | | 636.80560 | 1272.60393 | | -0.19 | 25.67 | 12/20 |
| VDcGYPHVTPKEcNNRGccFDSR | | | 45.58 | | 1.005E8 | | 707.79956 | 2828.17641 | | 0.25 | 20.74 | 37/132 |
| VDcGYPHVTPKEcNnRGccFDSR | | | 28.59 | | 5.318E7 | | 566.63788 | 2829.16029 | | 0.20 | 21.84 | 39/176 |
| EcNNRGccFDSR | | | 53.20 | |  | | 525.53333 | 1574.58542 | | -2.51 | 25.16 | 14/44 |
| DRVDcGYPHVTPK | | | 27.98 | | 1.422E8 | | 386.68784 | 1543.72951 | | -1.77 | 18.21 | 25/72 |
| EcNNRGccFDSR | | | 52.73 | |  | | 525.53345 | 1574.58579 | | -2.28 | 21.61 | 15/44 |
| EcNNRGccFDSR | | | 42.99 | |  | | 525.53363 | 1574.58634 | | -1.93 | 22.20 | 20/44 |
| EcNNRGccFDSR | | | 36.58 | |  | | 525.53302 | 1574.58451 | | -3.09 | 21.78 | 16/44 |
| VDcGYPHVTPKEcNNR | | | 28.70 | |  | | 487.22006 | 1945.85842 | | -3.08 | 23.24 | 30/90 |
| GccFDSR | | | 45.77 | |  | | 451.16821 | 901.32915 | | 0.01 | 21.37 | 10/12 |
| GccFDSR | | | 38.94 | |  | | 451.16776 | 901.32823 | | -1.00 | 21.88 | 10/12 |
| VDcGYPHVTPKEcnNRGccFDSR | | | 28.59 | | 5.318E7 | | 566.63788 | 2829.16029 | | 0.20 | 21.84 | 38/176 |
| EcNNRGccFDSR | | | 67.45 | | 1.420E8 | | 787.79816 | 1574.58904 | | -0.21 | 17.45 | 12/22 |
| VDcGYPHVTPK | | | 66.91 | |  | | 636.80444 | 1272.60161 | | -2.02 | 23.90 | 13/20 |
| GccFDSR | | | 39.37 | |  | | 451.16809 | 901.32891 | | -0.26 | 18.06 | 9/12 |
| EcNNRGccFDSR | | | 58.39 | |  | | 525.53418 | 1574.58799 | | -0.88 | 20.55 | 17/44 |
| EcNNRGccFDSR | | | 58.92 | |  | | 525.53278 | 1574.58377 | | -3.56 | 24.02 | 16/44 |
| GccFDSR | | | 34.30 | | 2.239E9 | | 451.16733 | 901.32738 | | -1.95 | 17.03 | 10/12 |
| VDcGYPHVTPKEcNNRGccFDSR | | | 36.28 | | 1.005E8 | | 707.79907 | 2828.17446 | | -0.44 | 21.02 | 35/132 |
| GccFDSR | | | 50.33 | |  | | 451.16803 | 901.32878 | | -0.40 | 20.11 | 10/12 |
| GccFDSR | | | 44.24 | |  | | 451.16782 | 901.32836 | | -0.87 | 22.08 | 9/12 |
| VDcGYPHVTPKEcNNR | | | 79.66 | | 1.532E7 | | 973.43622 | 1945.86516 | | 0.39 | 18.70 | 14/30 |
| GccFDSR | | | 52.27 | | 2.239E9 | | 451.16724 | 901.32720 | | -2.16 | 16.01 | 10/12 |
| VDcGYPHVTPKEcNNR | | | 31.33 | | 2.447E6 | | 487.21951 | 1945.85622 | | -4.21 | 25.27 | 29/90 |
| VDcGYPHVTPKEcNnRGccFDSR | | | 23.52 | | 5.318E7 | | 566.64001 | 2829.17097 | | 3.97 | 22.06 | 42/176 |
| VDcGYPHVTPK | | | 39.91 | | 9.774E6 | | 636.80560 | 1272.60393 | | -0.19 | 25.81 | 13/20 |
| GccFDSR | | | 33.38 | |  | | 451.16818 | 901.32909 | | -0.06 | 21.04 | 9/12 |
| GccFDSR | | | 47.94 | |  | | 451.16760 | 901.32793 | | -1.34 | 21.58 | 9/12 |
| VDcGYPHVTPKEcnNRGccFDSR | | | 23.52 | | 5.318E7 | | 566.64001 | 2829.17097 | | 3.97 | 22.06 | 42/176 |
| EcNNRGccFDSR | | | 52.73 | | 1.420E8 | | 787.79767 | 1574.58806 | | -0.83 | 15.94 | 9/22 |
| GccFDSR | | | 34.75 | |  | | 451.16751 | 901.32775 | | -1.55 | 23.84 | 9/12 |
| EcNNRGccFDSR | | | 56.00 | |  | | 787.79694 | 1574.58660 | | -1.76 | 19.40 | 9/22 |
| GccFDSR | | | 50.96 | |  | | 451.16870 | 901.33013 | | 1.09 | 20.86 | 10/12 |
| EcNNRGccFDSR | | | 26.11 | |  | | 525.53394 | 1574.58725 | | -1.35 | 14.68 | 13/44 |
| EcNNRGccFDSR | | | 42.44 | | 1.420E8 | | 787.79828 | 1574.58928 | | -0.06 | 16.79 | 10/22 |
| VDcGYPHVTPKEcNNR | | | 46.49 | | 1.532E7 | | 973.43591 | 1945.86455 | | 0.07 | 18.60 | 8/30 |
| GccFDSR | | | 45.01 | |  | | 451.16815 | 901.32903 | | -0.12 | 22.62 | 9/12 |
| GccFDSR | | | 44.24 | | 2.952E5 | | 451.16739 | 901.32750 | | -1.82 | 13.92 | 8/12 |
| VDcGYPHVTPKEcNNR | | | 32.04 | |  | | 487.22015 | 1945.85879 | | -2.89 | 24.11 | 27/90 |
| GccFDSR | | | 32.10 | |  | | 451.16864 | 901.33000 | | 0.96 | 23.21 | 10/12 |
| EcNNRGccFDSR | | | 40.83 | |  | | 787.79736 | 1574.58745 | | -1.22 | 19.25 | 8/22 |
| GccFDSR | | | 49.11 | |  | | 451.16766 | 901.32805 | | -1.21 | 19.07 | 11/12 |
| DRVDcGYPHVTPK | | | 43.08 | | 6.530E7 | | 772.36804 | 1543.72881 | | -2.23 | 18.47 | 12/24 |
| DRVDcGYPHVTPK | | | 35.29 | | 6.530E7 | | 772.36951 | 1543.73174 | | -0.33 | 19.69 | 9/24 |
| EcNNRGccFDSR | | | 84.61 | | 1.420E8 | | 787.79932 | 1574.59136 | | 1.26 | 16.35 | 9/22 |
| GccFDSR | | | 40.25 | |  | | 451.16888 | 901.33049 | | 1.50 | 25.74 | 8/12 |
| VDcGYPHVTPKEcNNR | | | 100.77 | | 1.532E7 | | 973.43652 | 1945.86577 | | 0.70 | 17.80 | 11/30 |
| GccFDSR | | | 23.85 | |  | | 451.16830 | 901.32933 | | 0.21 | 25.97 | 9/12 |
| EcNNRGccFDSR | | | 26.11 | | 8.152E8 | | 525.53363 | 1574.58634 | | -1.93 | 14.82 | 12/44 |
| EcNNRGccFDSR | | | 45.70 | |  | | 787.80005 | 1574.59282 | | 2.19 | 18.93 | 8/22 |
| VDcGYPHVTPKEcNNR | | | 42.63 | | 1.532E7 | | 973.43616 | 1945.86504 | | 0.32 | 18.37 | 9/30 |
| GccFDSR | | | 33.82 | | 1.008E6 | | 451.16684 | 901.32640 | | -3.04 | 24.86 | 8/12 |
| EcNNRGccFDSR | | | 32.48 | | 1.420E8 | | 787.79742 | 1574.58757 | | -1.14 | 14.90 | 9/22 |
| EcNNRGccFDSR | | | 31.10 | | 1.420E8 | | 787.79883 | 1574.59038 | | 0.64 | 17.26 | 6/22 |
| VDcGYPHVTPKEcNNR | | | 73.45 | | 5.204E8 | | 649.29205 | 1945.86161 | | -1.44 | 17.27 | 22/60 |
| EEYVGLSANQcAVPAKDRVDcG  YPHVTPK | | | 42.58 | | 1.728E8 | | 815.89197 | 3260.54604 | | -0.12 | 26.09 | 32/168 |
| GccFDSR | | | 22.77 | |  | | 451.16797 | 901.32866 | | -0.53 | 25.44 | 10/12 |
| EcNNRGccFDSR | | | 39.81 | |  | | 787.79913 | 1574.59099 | | 1.03 | 18.80 | 8/22 |
| EEYVGLSANQcAVPAKDRVDcG  YPHVTPK | | | 16.24 | | 1.728E8 | | 815.89197 | 3260.54604 | | -0.12 | 25.10 | 30/168 |
| EEYVGLSANQcAVPAKDRVDcG  YPHVTPK | | | 43.04 | | 1.728E8 | | 815.89233 | 3260.54751 | | 0.33 | 25.75 | 31/168 |
| VDcGYPHVTPK | | | 9.06 | |  | | 636.80420 | 1272.60112 | | -2.40 | 17.66 | 6/20 |

| Sample ID | TFF3 | | | | | |  |  | CD147 | | | | | |
| --- | --- | --- | --- | --- | --- | --- | --- | --- | --- | --- | --- | --- | --- | --- |
|  | TFF3-His_6_ | |  | | Resin | |  |  | TFF3-His_6_ | |  | | Resin | |
|  | # PSM | Area | | # PSM | | Area |  |  | # PSM | Area | | # PSM | | Area |
| 005 | 45 | 1.56E+09 | | N.D. | | N.D. |  |  | 29 | 2.03E+09 | | 5 | | 2.17E+07 |
| 484 | 44 | 5.91E+08 | | N.D. | | N.D. |  |  | 5 | 3.42E+08 | | N.D. | | N.D. |
| 488 | 14 | 2.04E+08 | | N.D. | | N.D. |  |  | 1 | 1.15E+07 | | N.D. | | N.D. |
| 489 | 24 | 9.86E+07 | | N.D. | | N.D. |  |  | 3 | 1.79E+07 | | N.D. | | N.D. |
| 500 | 31 | 4.48E+08 | | N.D. | | N.D. |  |  | 7 | 2.46E+08 | | 3 | | N.D. |
| 502 | 30 | 3.90E+08 | | N.D. | | N.D. |  |  | 19 | 7.65E+08 | | N.D. | | N.D. |
| 520 | 14 | 4.45E+08 | | N.D. | | N.D. |  |  | 2 | 5.73E+08 | | N.D. | | N.D. |

**Supplementary Table 4. Summary of LC/MS-MS analysis of CD147-TFF3 interaction.**

His pull-down was performed to identify TFF3-interacting proteins, where His_6_-tagged TFF3 protein was bait and the lysate from the fresh CRC tissues was prey. The eluted proteins were identified by mass-spectrometry. # PSM, the number of peptide spectrum matches.

| **Supplementary Table 5. Proteomics analysis of differentially expressed proteins.** | | | |
| --- | --- | --- | --- |
| A_vs_B compare | A_and_B common | A above | A below |
| Group_T_vs_Group_C | 4230 | 21 | 24 |
| Group_O_vs_Group_C | 4230 | 115 | 118 |
| Group T: TFF3 treatment; Group C: control; Group O: TFF3 overexpression. FC≥1.3, p<0.05 by two-tailed Student’s t test. | | | |

**Supplementary Table 6. Correlation between TFF3 and PTGS2 expression in colon cancer tissues.**

| TFF3  expression | PTGS2 expression | | | | Total, *N* |
| --- | --- | --- | --- | --- | --- |
|  | - | + | ++ | +++ |  |
| - | 4 | 18 | 6 | 1 | 29 |
| + | 2 | 13 | 39 | 7 | 61 |
| ++ | 1 | 10 | 12 | 5 | 28 |
| +++ | 0 | 2 | 5 | 3 | 10 |
| Total, *N* | 7 | 43 | 62 | 16 | 128 |

Using the Spearman correlation analysis, ρ=0.314, p<0.001

**Supplementary Table 7. Correlation between TFF3 and CD147 expression in colon cancer tissues.**

| CD147 expression | TFF3 expression | | | | Total, *N* |
| --- | --- | --- | --- | --- | --- |
|  | - | + | ++ | +++ |  |
| - | 5 | 7 | 2 | 2 | 16 |
| + | 9 | 22 | 6 | 0 | 37 |
| ++ | 3 | 5 | 2 | 0 | 10 |
| Total, *N* | 17 | 34 | 10 | 2 | 63 |

Using the Spearman correlation analysis, ρ=-0.031, p=0.812

**Supplementary Table 8. Serum levels of TFF3 and CD147 in CRC patients**

| Patient ID | Serum CD147 (pg/mL) | Serum TFF3 (pg/mL) |
| --- | --- | --- |
| 1001 | 194.45 | 3466.25 |
| 1002 | 146.72 | 2697.5 |
| 503 | 330.83 | 5491.25 |
| 1004 | 208.09 | 4947.5 |
| 1005 | 337.65 | 7347.5 |
| 506 | 245.59 | 2710 |
| 521 | 204.68 | 2847.5 |
| 1008 | 277.98 | 2528.75 |
| 1009 | 257.53 | 4535 |
| 1010 | 221.73 | 3953.75 |
| 1011 | 143.31 | 5278.75 |
| 1012 | 151.83 | 7785 |
| 1013 | 252.41 | 8947.5 |
| 526 | 116.03 | 4766.25 |
| 528 | 197.86 | 1641.25 |
| 1016 | 173.99 | 6491.25 |
| 1017 | 117.74 | 1360 |
| 1018 | 146.72 | 4768.33 |
| 1019 | 305.26 | 4897.5 |
| 535 | 225.14 | 5610 |
| 672 | 87.05 | 2310 |
| 1022 | 184.22 | 6116.25 |
| 1023 | 196.16 | 3085 |
| 1024 | 306.96 | 7397.5 |
| 541 | 400.72 | 3441.25 |
| 1026 | 197.86 | 6460 |
| 1027 | 116.03 | 5241.25 |
| 1028 | 187.63 | 2403.75 |
| 1029 | 220.02 | 2816.25 |
| 1030 | 141.6 | 3241.25 |
| 547 | 175.7 | 4360 |
| 548 | 160.36 | 2041.25 |
| 1033 | 221.73 | 9110 |
| 550 | 204.68 | 2185 |
| 1035 | 153.54 | 2741.25 |
| 552 | 179.11 | 2753.75 |
| 1037 | 211.5 | 4047.5 |
| 1038 | 288.21 | 4141.25 |
| 1039 | 175.7 | 1478.75 |
| 1040 | 148.42 | 1872.5 |
| 1041 | 134.78 | 2178.75 |
| 1042 | 185.93 | 1941.25 |
| 1043 | 259.23 | 4347.5 |
| 1044 | 201.27 | 4760 |
| 1045 | 133.08 | 1147.5 |
| 1046 | 191.04 | 1360 |
| 1047 | 170.58 | 1660 |
| 1048 | 255.82 | 3297.5 |
| 565 | 238.77 | 6139.17 |
| 1050 | 168.88 | 4664.17 |
| 567 | 185.93 | 3318.33 |
| 1052 | 221.73 | 3647.5 |
| 1053 | 218.32 | 2630.83 |
| 1054 | 199.56 | 1597.5 |
| 571 | 179.11 | 2497.5 |
| 1056 | 143.31 | 1201.67 |
| 1057 | 209.79 | 5072.5 |
| 1058 | 199.56 | 4122.5 |
| 1059 | 155.24 | 1355.83 |
| 1060 | 146.72 | 2393.33 |
| 1061 | 114.33 | 3722.5 |
| 579 | 167.17 | 2439.17 |
| 1063 | 153.54 | 1626.67 |
| 1064 | 201.27 | 4164.17 |
| 1065 | 179.11 | 1268.33 |
| 1066 | 179.11 | 6260 |
| 1067 | 228.55 | 4355.83 |
| 590 | 156.95 | 2043.33 |
| 1069 | 165.47 | 5764.17 |
| 1070 | 175.7 | 2851.67 |
| 1071 | 177.4 | 1680.83 |
| 1072 | 88.76 | 3476.67 |
| 1073 | 138.19 | 7514.17 |
| 1074 | 221.73 | 885 |
| 1075 | 221.73 | 2564.17 |
| 1076 | 204.68 | 2635 |
| 1077 | 194.45 | 2410 |
| 1078 | 274.57 | 2068.33 |
| 1079 | 267.75 | 1355.83 |
| 1080 | 141.6 | 5072.5 |
| Mean (pg/mL) | 195.8 | 3660.0 |
| SD (pg/mL) | 56.8 | 1930.0 |
| Pearson r | 0.228^a)^ | |

^a)^p=0.042 by Pearson correlation, n=80.

**Supplementary Table 9. Summary of immunohistochemical analysis of surgical specimens of human colorectal cancer**

| **Total** | **TFF3^+^** | **CD147^+^** | **TFF3^+^CD147^+^** | **TFF3^-^CD147^-^** |
| --- | --- | --- | --- | --- |
| 886 | 579  (65.35%) | 687  (77.54%) | 489 (55.19%) | 98 (11.07%) |
| pTNM Stage (n) |  |  |  |  |
| 0 (32) | 17 (53.13%) | 14 (43.75%) | 7 (21.88%) | 8 (25.00%) |
| Ⅰ (121) | 72 (59.50%) | 86 (71.07%) | 54 (44.63%) | 17 (14.05%) |
| Ⅱ (328) | 205 (62.50%) | 262 (79.88%) | 171 (52.13%) | 33 (10.06%) |
| Ⅲ (382) | 267 (69.90%) | 305 (79.84%) | 240 (62.83%) | 39 (10.21%) |
| Ⅳ (23) | 18 (78.26%) | 20 (86.96%) | 17 (73.91%) | 1 (4.35%) |
| Spearman r  (p value) | 0.105  (0.002) | 0.106  (0.002) | 0.18  (<0.001) | -0.063  (0.061) |
| p value | 0.05 | <0.001 | <0.001 | 0.032 |

The expression patterns of TFF3 and CD147 in 886 samples of colorectal cancer were determined and summarized.

| Supplementary Table 10. Primer sequences | | |
| --- | --- | --- |
| Real-time RT-PCR primers | | |
| *PTGS2* | Forward | 5’-CTGGCGCTCAGCCATACAG-3’ |
|  | Reverse | 5’-CGCACTTATACTGGTCAAATCCC-3’ |
| *CABC1* | Forward | 5’-GCTTGACAAGGCCTTTAGCC-3’ |
|  | Reverse | 5’-GAGGCGGCTAGAAGGTGAC-3’ |
| *PTGER1* | Forward | 5’-AGCTTGTCGGTATCATGGTGG-3’ |
|  | Reverse | 5’-AAGAGGCGAAGCAGTTGGC-3’ |
| *PTGER 2* | Forward | 5’-CGATGCTCATGCTCTTCGC-3’ |
|  | Reverse | 5’-GGGAGACTGCATAGATGACAGG-3’ |
| *PTGER 3* | Forward | 5’-CGCCTCAACCACTCCTACAC-3’ |
|  | Reverse | 5’-GACACCGATCCGCAATCCTC-3’ |
| *PTGER 4* | Forward | 5’-CCGGCGGTGATGTTCATCTT-3’ |
|  | Reverse | 5’-CCCACATACCAGCGTGTAGAA-3’ |
| *GAPDH* | Forward | 5’-GCACCGTCAAGGCTGAGAAC-3’ |
|  | Reverse | 5’-TGGTGAAGACGCCAGTGGA-3’ |
| *PTGS2*  (ChIP) | Forward | 5’-AGCTTCCTGGGTTTCCGATT-3’ |
|  | Reverse | 5’-CCCCACAAATTTTTCCCTCCTC-3’ |
| Mutant primers | | |
| STAT3 binding site 1 | Forward | 5'-ccccttaaaaaaattgcggccgcccggtgggggcaggg-3' |
|  | Reverse | 5'-ccctgcccccaccgggcggccgcaatttttttaagggg-3' |
| STAT3 binding site 2 | Forward | 5'-ccctcctctccccttaaagccattgcgtaagcccggtgg-3' |
|  | Reverse | 5'-ccaccgggcttacgcaatggctttaaggggagaggaggg-3' |
| STAT3 binding sites 1 and 2 | Forward | 5'-CCTCCTCTCCCCTTAAAGCCATTGCGGCCGCCCGGTG-3' |
|  | Reverse | 5'-CACCGGGCGGCCGCAATGGCTTTAAGGGGAGAGGAGG-3' |
| NF κB binding site | Forward | 5'-GGGAGCAGAGGGGGTAGGAACCACTCTCCTGTCTGAT-3' |
|  | Reverse | 5'-ATCAGACAGGAGAGTGGTTCCTACCCCCTCTGCTCCC-3' |
| CD147^R54A^ | Forward | 5'-ccccttcagccaggcgtgccctgtgacc-3' |
|  | Reverse | 5'-ggtcacagggcacgcctggctgaagggg-3' |
| CD147^W82A^ | Forward | 5'-caggagtactctcccgcctggtcgtcggagtc-3' |
|  | Reverse | 5'-gactccgacgaccaggcgggagagtactcctg-3' |
| CD147^E84A^ | Forward | 5'-gacgcaggagtacgctccccactggtc-3' |
|  | Reverse | 5'-gaccagtggggagcgtactcctgcgtc-3' |
| CD147^G95A^ | Forward | 5'-atgttggccgtggccatgggctcgg-3' |
|  | Reverse | 5'-ccgagcccatggccacggccaacat-3' |
| CD147^T121A^ | Forward | 5'-ccagcatggccgcctccccctcgtt-3' |
|  | Reverse | 5'-aacgagggggaggcggccatgctgg-3' |
| CD147^M123A^ | Forward | 5'-cttgcagaccagcgcggccgtctccccc-3' |
|  | Reverse | 5'-gggggagacggccgcgctggtctgcaag-3' |
| CD147^V131A^ | Forward | 5'-caagtcagagtccgcgccacctgtcactg-3' |
|  | Reverse | 5'-cagtgacaggtggcgcggactctgacttg-3' |
| CD147^R166A^ | Forward | 5'-tgtgtagctctgacgcgccctgcgaggaac-3' |
|  | Reverse | 5'-gttcctcgcagggcgcgtcagagctacaca-3' |
| CD147^H170A^ | Forward | 5'-gttcaggttctcaatggctagctctgaccggccc-3' |
|  | Reverse | 5'-gggccggtcagagctagccattgagaacctgaac-3' |
| Genotyping primers | | |
| *CD147*^f/f^ | *CD147*-F1 | 5'-CTGGAACTCCTAGCAATC-3' |
|  | *CD147*-R1 | 5'-TGGGAAAGGGTTAGTAC-3' |
| Villin^Cre^ | Cre-F | 5’-gtgtttggtttggtttcctctgcataaga-3’ |
|  | Cre-R | 5’-gcaggcaaattttggtgtacggtca-3’ |
| *Tff3*^-/-^ | *Tff3*-R | 5’-GGAACTGTGCCCTGGAAGTGTAAGT-3’ |
|  | *Tff3*-F1 | 5’-GCTCAGGTCTAGAAACCAGAGGTACT-3’ |
|  | *Tff3*-F2 | 5’-CCCTTTCCTTCCTTCCTTCCCAGAAT-3’ |
